# The Effect of Scandium Ternary Intergrain Precipitates in Al-Containing High-Entropy Alloys

**DOI:** 10.3390/e20070488

**Published:** 2018-06-22

**Authors:** Sephira Riva, Shahin Mehraban, Nicholas P. Lavery, Stefan Schwarzmüller, Oliver Oeckler, Stephen G. R. Brown, Kirill V. Yusenko

**Affiliations:** 1 College of Engineering, Swansea University, Swansea SA1 8EN, Wales, UK; 2 Faculty of Chemistry and Mineralogy, Institute for Mineralogy, Crystallography and Materials Science, Leipzig University, Scharnhorststr. 20, 04275 Leipzig, Germany; 3 Institute of Solid State Chemistry, Pervomaiskaia str. 91, 620990 Ekaterinburg, Russia

**Keywords:** high-entropy alloys, in situ X-ray diffraction, grain refinement, thermoelectric properties, scandium effect

## Abstract

We investigate the effect of alloying with scandium on microstructure, high-temperature phase stability, electron transport, and mechanical properties of the Al_2_CoCrFeNi, Al_0.5_CoCrCuFeNi, and AlCoCrCu_0.5_FeNi high-entropy alloys. Out of the three model alloys, Al_2_CoCrFeNi adopts a disordered CsCl structure type. Both of the six-component alloys contain a mixture of body-centered cubic (*bcc*) and face centered cubic (*fcc*) phases. The comparison between in situ high-temperature powder diffraction data and ex situ data from heat-treated samples highlights the presence of a reversible *bcc* to *fcc* transition. The precipitation of a MgZn_2_-type intermetallic phase along grain boundaries following scandium addition affects all systems differently, but especially enhances the properties of Al_2_CoCrFeNi. It causes grain refinement; hardness and electrical conductivity increases (up to 20% and 14% respectively) and affects the CsCl-type → *fcc* equilibrium by moving the transformation to sensibly higher temperatures. The maximum dimensionless thermoelectric figure of merit (*ZT*) of 0.014 is reached for Al_2_CoCrFeNi alloyed with 0.3 wt.% Sc at 650 °C.

## 1. Introduction

High-Entropy Alloys (HEAs) are defined according to the atomic percentage of their principal elements, between 5 and 35 at %, or according to their configurational entropy at random state, ΔS > 1.5 R (R = 8.314 J·K^−1^·mol^−1^). Their high compositional complexity draws a hyper-dimensional space whose limits have yet to be explored, for its investigation has mostly focused on equiatomic or near-equiatomic compositions [1,2]. In fact, the study of HEAs is driven by the search of new single-phase systems, whose formation is opposed by the diverse mixing enthalpies, atomic size, and valence electron concentration of the constituent elements. Consequently, single-phase HEAs are rare, whereas systems consisting of multiple solid solutions and ordered intermetallic phases are more common [3].

Following the pioneering publications in 2004 by Yeh [4] and Cantor [5], HEAs have attracted growing research interest due to their outstanding mechanical and thermal properties; including high compression yield, fracture strength, ductility and toughness, as well as extreme corrosion, wear and fatigue resistance. HEAs have thus been proposed as candidates for applications in which high temperature stability is pivotal; e.g., as replacements for conventional binders after liquid-phase or spark-plasma sintering, for liquefied gas storage and for high-temperature thermoelectrics [1,6]. Thermoelectric properties can be tuned with respect to valence electron concentration (VEC) and a dimensionless thermoelectric figure of merit (*ZT*) of 0.012 at 505 °C was reached for Al_2_CoCrFeNi. The phase evolution of HEAs has been extensively studied both ex situ and in situ [7,8,9,10,11].

Following a traditional trend in alloy development, HEAs have seen the addition of selected secondary phases to further tune their mechanical properties. This approach has led to the development of a new class of metal-matrix composites, containing oxides [12], silicon carbide [13] or nano-diamonds [14]. Alloying with elements in low concentrations, on the other hand, has resulted in the precipitation of intermetallic compounds in the matrix phase. However, while intermetallics deeply affect yield strength, hardness, tensile properties, and matrix stabilization—due to the competition between mixing enthalpy of atom pairs and mixing entropy—their proper distribution, size, shape, and volume fraction represent a cause for concern [1]. The synthesis of precipitation-hardened HEAs following the introduction of intermetallics has proven largely unsuccessful when binary compounds are concerned, and no studies have been performed on the formation of stable ternary inclusions as pinning centers in complex multi-principal component alloys [15,16,17]. This is mostly due to the difficulties in choosing appropriate alloying elements. Their selection should be guided by the following considerations: miscibility for most or all HEA constitutive elements in liquid state, low formation enthalpy for ternary compounds and crystallization in common structure types (e.g., σ-phase, Laves phase).

We recently highlighted the compound-forming ability of scandium and its outstanding effect on the mechanical properties of multicomponent alloys [18]. Scandium forms over three hundred binary and ternary phases with most elements of the periodic table, many of which crystallize in highly symmetrical structures (e.g., space groups *Pm*$\bar{3}$*m*, *Fm*$\bar{3}$*m*, *P*$6_{3}$*/mmc*). Scandium-based intermetallics (i.e., Al_3_Sc, *V*- and *W*-phases) are responsible for the enhanced properties of several commercial aluminum alloys and newly developed multicomponent systems [19]. These features make scandium a perfect candidate to achieve precipitation-hardened HEAs. Moreover, its low density makes it ideal in combination with HEA based on 3*d*-elements, which make up to 85% of the known systems [1]. The Al_x_CoCrCu_y_FeNi HEA can be considered a model alloy. The nature of the solid solution can be tuned by changing the aluminum and copper content, since Al and Cu act as *bcc*- and *fcc*-stabilizers respectively [20]. Thus, Al_2_CoCrFeNi and AlCoCrCu_0.5_FeNi have been widely reported as pure *bcc* phases, while Al_0.5_CoCrCuFeNi as purely *fcc*-structured [21,22,23].

We herein report on the effects of 0.3–5 wt.% scandium addition to the microstructure, mechanical and transport properties, thermal stability, and phase evolution upon temperature of the model Al_2_CoCrFeNi, AlCoCrCu_0.5_FeNi, and Al_0.5_CoCrCuFeNi HEAs. We show that scandium forms the same stable ternary intermetallic in all three alloys, but that the compound interacts differently with *bcc*- and *fcc*-structured alloys.

## 2. Materials and Methods

The target alloys were prepared using induction melting from pure metallic powders, in a BN crucible in an Ar-filled Customised DAB01 glove-box (Saffron Scientific Equipment Limited, Knaresborough, UK). Complete melting of the samples was achieved above 1300 °C. After 5 min at the melting temperature, the sample was cooled down naturally to room temperature. The samples were re-melted three times to assure homogeneity. A brief rationale of the synthesized specimens and their performed analysis is reported in Supplementary Materials Table S1.

Differential scanning calorimetry (DSC) measurements were performed on small pieces of sintered samples (50 mg) placed in an Al_2_O_3_ crucible and heated in a Netzsch STA 449 F1 Jupiter. Heating (Selb, Germany) and cooling were performed in flowing Ar gas with a temperature ramp of 10 K·min^−1^ from 35 to 1300 °C.

The transition temperatures highlighted by DSC were used to decide annealing conditions. Samples were heat-treated above their first reversible or irreversible transition temperature with the following specifics: Al_2_CoCrFeNi, Al_0.5_CoCrCuFeNi and AlCoCrCu_0.5_FeNi at 850 °C, 12 h; Al_2_CoCrFeNi + 3 wt.% Sc at 900 °C, 12 h; Al_0.5_CoCrCuFeNi + 3 wt.% Sc and AlCoCrCu_0.5_FeNi + 3 wt.% Sc at 930 °C for 6 h. During annealing, pellets of each sample were sealed in a silica tube under vacuum (<10^−7^ Pa) and heated in a furnace. After annealing, the tubes were quenched in ice-cold water.

For microstructure and elemental analysis, all samples were mounted in carbonized resin, ground and polished using MetaDiTM Supreme Polycrystalline Diamond Suspension (1 μm) (Coventry, UK).

The morphology and elemental compositions were analyzed using a Hitachi S-4800 Field Emission scanning-electron microscope (SEM) equipped with energy dispersive X-ray (EDX) analyzer (Tokio, Japan). The average elemental composition was obtained from 2.5 mm maps (Table S2).

The Vickers hardness was measured on a WilsonR VH3100 Automatic Knoop/Vickers Hardness tester (Buehler, Lake Bluff, IL, USA); 25 individual points under a 9.81 N (1 kg) testing load were measured to get statistically significant results.

The density was measured according to Archimedes’ principle in water, in the ATTENSION equipment (Biolin Scientific, Stockholm, Sweden). Six measurements were taken for each sample to obtain statistically relevant results

The small punch tests were performed on discs of diameter 12.5 mm and thickness circa 0.8 mm. Measurements were performed with a properly modified Tinius Olsen H25KS Benchtop Tester (Salfords, UK). The setup included a lower die (diameter of 8 mm) and a punch (4 mm diameter). Each experiment was reproduced twice for statistical significance; however, since each specimen had a slightly different thickness, the final results were normalized for the standard 0.5 mm thickness as described in [24].

For powder X-ray diffraction (PXRD), samples were powdered using a Fritsch mini-mill Pulverisette 23 (Idar-Oberschtein, Germany) (steel vial and ball, 10 min at 50 rpm). PXRD data for the annealed powdered samples were collected at ID06B-LVP beam-line at the European Synchrotron Research Facility, ESRF (room temperature, λ = 0.22542 Å) using position sensitive detector. LaB_6_ (NIST SRM 660c) was used as external standard for calibration.

In situ high-temperature PXRD patterns were collected at the I-11 beam-line at the DIAMOND light source (λ = 0.494984 Å). LaB_6_ (NIST SRM 660c) was used as external standard for wavelength and sample to detector distance calibration. A wide-angle Mythen-2 Si position sensitive detector. The detector was moved at constant angular speed with 10 s scan time at each temperature and 60 s waiting time to let the temperature stabilize. The powdered alloys were sealed in a 0.5 mm silica glass capillary in vacuum and heated in the capillary furnace from 25 to 1200 °C with axial rotation [25]. In all samples, oxidation was detected above 1000 °C, which can be due to the reaction of metallic alloy with silica at high temperature, resulting in capillary destruction. In situ low-temperature PXRD profiles were collected at the P02.1 beam-line at the PETRA III synchrotron (λ = 0.207150 Å). LaB_6_ (NIST SRM 660c) was used as external standard for calibration. A wide-angle position sensitive detector based on Mythen-2 Si strip modules was used. The detector was moved at constant angular speed with 10 s scan time at each temperature and 60 s waiting time to let the temperature stabilize. The powdered alloys were sealed in 0.5 mm silica glass capillaries in vacuum and cooled in nitrogen flow from 300 to 100 K. Temperature was directly measured with a thermocouple during the experiment; error arising from the distance between sensor and capillary was estimated to be below 5%.

Temperature dependent PXRD patterns were analyzed using Powder3D software [26]. Phase composition has been verified using the Powder Diffraction File database [27]. Parametric sequential refinements were performed using the TOPAS 5.0 software [28]. Profile parameters for the Lorentzian function, cell parameters, and phase fractions were refined simultaneously for all phases.

The Seebeck coefficient S (µV·K^−1^) and electrical conductivity σ (kS·cm^−1^) were measured simultaneously under He atmosphere with a Linseis – Seebeck and Electric Resistivity Unit (LSR-3 1100, Linseis, Selb, Germany) four-point setup with PtRh/Pt and Pt contacts and a continuous reverse of the polarity of the thermocouples (bipolar setup, measurement current: 100 mA) using cuboid samples (ca. 8 × 2 × 3 mm). Three heating cycles up to 875 °C (10 K·min^−1^, 3 data points per temperature) were performed. Thermal diffusivity was measured up to 875 °C (heating/cooling rate 10 K·min^−1^) under He atmosphere with a Linseis LFA1000 (Selb, Germany) apparatus. Simultaneous heat loss and finite pulse corrections were applied using Dusza’s model [29]. Values were averaged from five measurement points at each temperature. For calculation of thermal conductivity κ, they were multiplied with the Dulong−Petit heat capacity C_p_ and the density as derived by the weight and the volume determined by Archimedes’ principle. The single values of each sample are given in Table S3. According to experimental C_p_ values of materials with similar compositions (e.g., 0.60 J·g^−1^·K^−1^ for AlCoCrFeNi at 25 °C) [30], the room temperature heat capacity of these materials is about 20% higher than the Dulong–Petit value of 0.49 J·g^−1^·K^−1^; this probably adds this uncertainty to the values of κ and thus *ZT*.

## 3. Results and Discussions

**Figure 1:**
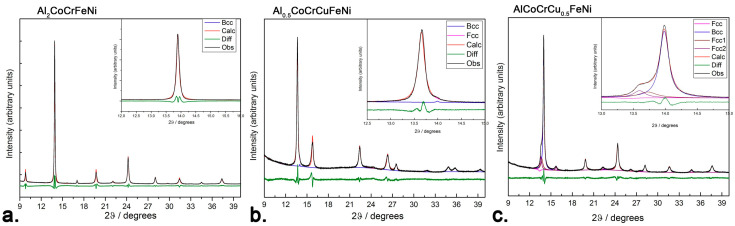
Powder X-ray diffraction (PXRD) Rietveld refinements performed from the DIAMOND light source (I11, λ = 0.494984 Å) of (**a**) Al_2_CoCrFeNi. The section between 12 and 16 degrees 2θ is enlarged to show the symmetric shape of the first reflection. (**b**) Al_0.5_CoCrCuFeNi. The section between 12 and 15 degrees 2θ is enlarged to show the asymmetry of the first reflection, which can only be fitted by taking into account a small amount of *bcc* phase. (**c**) AlCoCrCu_0.5_FeNi. The section between 13 and 15 degrees 2θ is enlarged to show the presence of an *fcc* phase.

Al_2_CoCrFeNi, Al_0.5_CoCrCuFeNi and AlCoCrCu_0.5_FeNi were synthesized via induction melting in the atomic compositions reported in the Table S1. While all three systems are widely reported as single-phase [1], refinements of PXRD data obtained with synchrotron radiation highlight the presence of a secondary *fcc* phase in the *bcc*-structured AlCoCrCu_0.5_FeNi alloy (Figure 1c) and of a very small secondary *bcc* phase in the mainly *fcc*-structured Al_0.5_CoCrCuFeNi alloy (Figure 1b). Unlike Al_0.5_CoCrCuFeNi and AlCoCrCu_0.5_FeNi, Al_2_CoCrFeNi appears to be a solid solution forming a disordered CsCl structure-type. (Figure 1a). This is consistent with a previously reported investigation on CoCrCuFeNi-based systems—which displayed a phase separation due to the positive mixing enthalpy between copper and other elements [31]—and with the Hume–Rotary classification maps in ref. [32].

The presence of the (100) diffraction line in the *bcc*-structured PXRD pattern indicates an ordered superstructure, generally attributed to Al and Ni ordering [22,33,34]. Lattice parameters of the as-cast alloys are the following: *a_B_*_2_ = 2.877(2) Å for Al_2_CoCrFeNi; *a_fcc_* = 3.601(1) Å for Al_0.5_CoCrCuFeNi and *a_bcc_* = 2.891(3) Å for AlCoCrCu_0.5_FeNi. All values are consistent with the literature within the experimental error [20,22,35,36,37]. Small differences between the reported results arise from the high sensitivity of the HEA to synthetic pathway and to minor compositional variations. Therefore, nominally equivalent starting materials can in turn display profoundly different crystal structures, microstructures, and phase transitions.

The microstructures and elemental distributions of as-cast Al_2_CoCrFeNi, Al_0.5_CoCrCuFeNi and AlCoCrCu_0.5_FeNi are reported by means of EDX element mapping in Figures S1–S3, respectively. Two phases are present in the as-cast AlCoCrCu_0.5_FeNi alloy, whose microstructure is characterized by a matrix and a circular secondary phase of darker color. Elemental distributions appear completely homogeneous (Figure S3). Annealing results in the growth of a darker secondary phase and the slight segregation of Al from the rest of the elements. The as-cast Al_0.5_CoCrCuFeNi HEA consists of dendritic-like and interdendritic-like regions, the first being richer in Co, Cr and Fe; and the latter Cu-rich (Figure S2). Both the dendrite-like and interdendrite-like matrix have been previously linked to a simple *fcc* phase, and copper segregation has been explained through its high mixing enthalpy with cobalt, chromium, iron, and nickel [20]. The microstructure changes drastically after annealing, as the two phases cannot be easily differentiated. Nevertheless, element segregation persists, with Cu and Al separating from Co, Cr, Fe, and Ni. The as cast Al_2_CoCrFeNi microstructure is dominated by large (∼100 μm) unstructured grains which, unlike previous studies, show no trace of non-equiaxed dendrites [21,37]. With respect to other elements, chromium segregation is clearly visible, though it can be reduced by annealing (see Figure S1). On the other hand, annealing causes the formation of a homogeneously dispersed secondary phase, which appears as black dots.

**Figure 2:**
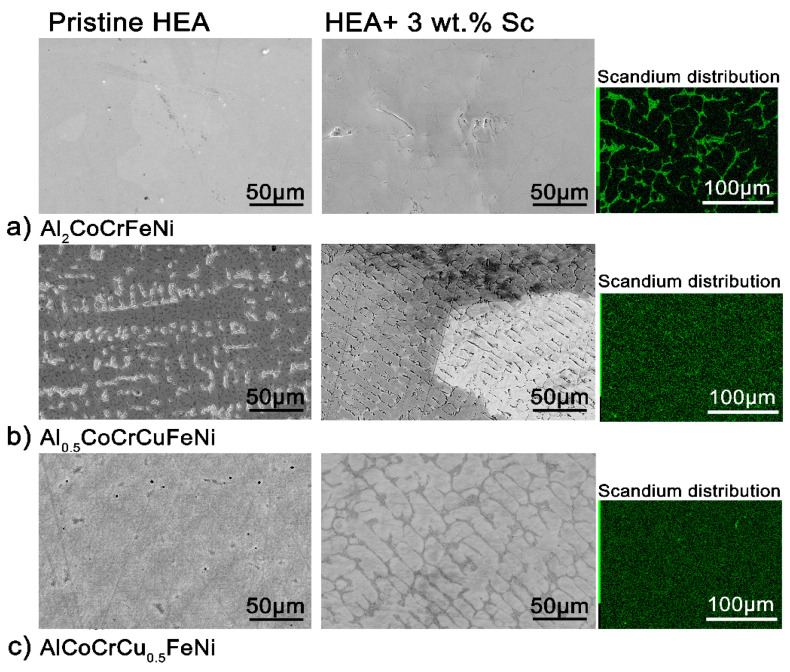
Overview of the effect of scandium addition on the microstructure of the three systems (scanning electron microscope-back scatter detector (SEM-BSE) images). **Left**: Microstructure of the as-cast (**a**) Al_2_CoCrFeNi, (**b**) Al_0.5_CoCrCuFeNi and (**c**) AlCoCrCu_0.5_FeNi High-Entropy Alloys (HEAs) before and after 3 wt.% scandium addition. **Right**: scandium distribution of the areas displayed in (**b**) according to energy dispersive X-ray (EDX) maps.

The microstructure of all alloys is strongly affected by even a 3 wt.% addition of scandium. In the case of Al_2_CoCrFeNi (Figure 2a, Figure S4), the large grains of homogeneous compositions are refined and scandium segregates in the inter-grain volume. The scandium-based intermetallic forming in the inter-granular region appears to contain all elements in the same relative fractions as the main phase—with the notable exception of chromium (Table S1). In Al_0.5_CoCrCuFeN (Figure 2b), scandium is dispersed more homogeneously and aids the formation of a globular microstructure. Nevertheless, most of it segregates in the inter-granular region, with copper- and nickel-rich areas (Figure S5). Lastly, following scandium addition the original columnar cellular microstructure of the AlCoCrCu_0.5_FeNi HEA turns into equiaxed non-dendritic-like grains (Figure 2c). The chemical inhomogeneity due to scandium segregation appears surprising, considering the strongly negative mixing enthalpy of the metal with iron, nickel, cobalt and aluminum [38,39,40]. The driving force of the segregation is thus the formation of the secondary phase.

**Figure 3:**
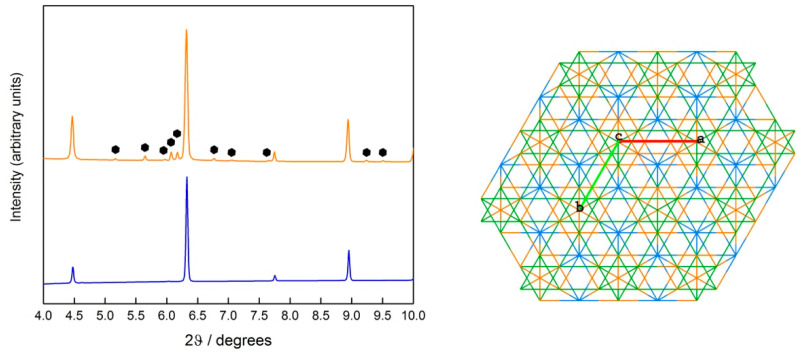
**Left**. PXRD profile (DIAMOND = 0.22542 Å) of the as-cast disordered CsCl-structured Al_2_CoCrFeNi alloy before (blue) and after (orange) a 3 wt.% Sc addition. The PXRD profile of the scandium phase is indexed with black hexagons. **Right**. Crystal structure of the hexagonal MgZn_2_-type intermetallic along the c-axis, depicted as its AlCuSc analogue (Al in green, Cu in orange and Sc in blue; a, b and c are cell axis).

The secondary phase is the same in all systems and can be indexed as a ternary intermetallic analogous of MgZn_2_-type (Figure 3). Compounds of this structure type have been reported for scandium with several metals, in compositions such as AlCuSc, AlCoSc, Al_1.06_Cr_0.94_Sc, AlFeSc, and AlNiSc [18]. As confirmed by elemental composition maps (Figures S4–S6), the ternary phase is a highly disordered structure containing all five elements and scandium.

Knowledge about scandium-containing ternary compounds is still fragmentary. Out of all the cited MgZn_2_-type intermetallic phases, only AlCuSc has been thoroughly investigated, due to its effect on the mechanical properties of Al-based alloys, as part of the so-called *W*-phase. In particular, it was shown that the microhardness of the *W*-phase is much higher than the one of the Al_3_Sc phase (5150–5170 MPa against 3900–4300 MPa) [18].

**Figure 4:**
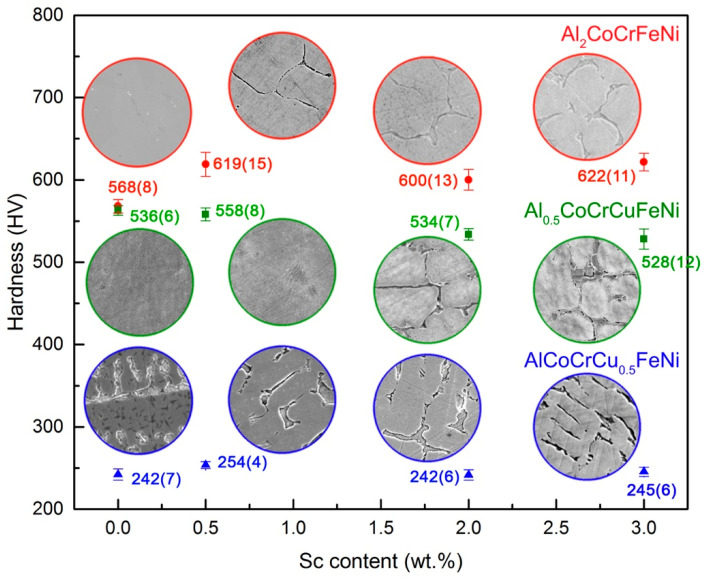
Vickers hardness values for Al_2_CoCrFeNi (red), Al_0.5_CoCrCuFeNi (green) and AlCoCrCu_0.5_FeNi (blue) HEAs with 0, 0.5, 2 and 3 wt.% Sc additions. Values are an average of 25 indentations at 1 HV. SEM images of the microstructures of all alloys are shown in circles of 30 μm diameter.

The formation of a very hard phase in the HEA matrix affects its mechanical properties. As shown in Figure 4, single phase *bcc* alloys are harder than duplex-structured and *fcc* alloys. This is hardly surprising, considering the stronger interatomic forces involved in the *bcc* vs. *fcc* packing of alloys. On the other hand, increasing scandium content in Al_0.5_CoCrCuFeNi does not affect hardness. Indeed, it is even detrimental to the hardness of the AlCoCrCu_0.5_FeNi alloy and is not accompanied by an increase in ductility (as shown by the disk punch tests presented in Figure S7). Only in the originally hard Al_2_CoCrFeNi HEA the formation of the intermetallic results in an increase in hardness. The addition of 0.5 wt.% Sc causes a 20% hardness enhancement, as well as visible grain refinement. Disc punch tests performed on the HEA with 0, 0.5 and 2 wt.% scandium additions show a decisive increment in brittleness, proportional to the concentration of scandium (Figure S8).

**Figure 5:**
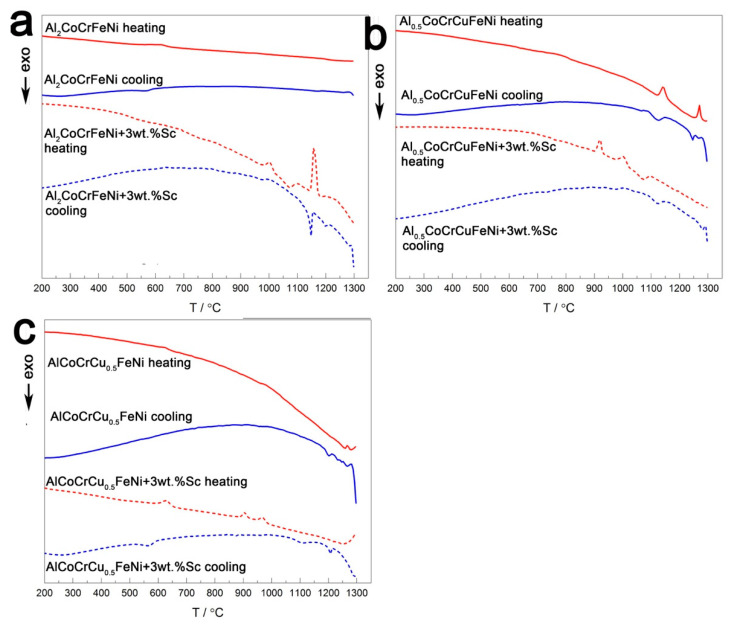
Differential scanning calorimetry (DSC) of (**a**) Al_2_CoCrFeNi, (**b**) Al_0.5_CoCrCuFeNi and (**c**) AlCoCrCu_0.5_FeNi with (dotted line) and without (solid line) 3 wt.% scandium. The second heating/cooling cycle is reported for each specimen, in red and blue respectively.

Differential scanning calorimetry (DSC) was performed on alloys from room temperature to 1300 °C with a 10 K·min^−1^ heating rate. The second cycle of heating and cooling, which is less influenced by effects of the synthetic route on the specimens—i.e., internal stress-strain, magnetic ordering—is reported in Figure 5.

The DSC profile of Al_2_CoCrFeNi (Figure 5a) has a sigmoid-like deviation between 600 and 700 °C. In a theoretical work, Gao associates this feature with the transition *bcc*_1_ *+ bcc*_2_ *+* CsCl → *bcc+*CsCl, but this is incompatible with the crystal structure of the Al_2_CoCrFeNi alloy as per Figure 1a. The divergence between the results reported here and Gao’s interpretation might arise from the profound differences in the crystal structures of the nominally equivalent starting material [41]. To unequivocally identify the cause of the transition, a more detailed investigation of the effect of temperature on phase stability is needed. The corresponding Sc-containing sample displays a sharp reversible peak at 1150 °C, probably corresponding to the melting and crystallization of the intermetallic phase, preceded by irreversible peaks. These two endothermic peaks, located at 906 °C and 966 °C, might correspond to phase transitions occurring in the scandium-phase. The sigmoid-like deviation clearly visible in the pristine alloy is hardly distinguishable from the background line in the Sc-containing specimen but is located at higher temperature (between 700 and 800 °C).

Al_0.5_CoCrCuFeNi shows a slight reversible peak centered at 760 °C, as well as two reversible peaks above 1150 °C (Figure 5b). In previously reported DSC curves, the two endothermic peaks of Al_0.5_CoCrCuFeNi at 1140 and 1270 °C have been linked to the melting of interdendritic-like and dendritic-like material, respectively. The results of Jones et al. highlight a third reversible peak, appearing at 850 °C and related to the dissolution and recrystallization of the L1_2_ phase, which might form during prolonged heat treatment below 850 °C and is dependent from the sample cooling rate [42,43]. Its absence is indicative of the purity of the as-cast *fcc* sample, which is confirmed by high-resolution PXRD data (Figure 1b). The scandium containing specimen displays three irreversible signals upon heating (the first two, endothermic, at 921 and 1000 °C; the latter, exothermic, at 1073 °C). Upon cooling, the Sc-containing specimen behaves very similarly to its corresponding pristine alloy. The irreversible transitions occurring in the sample might thus relate solely to the intermetallic, and have little impact on the matrix.

Finally, AlCoCrCu_0.5_FeNi displays a reversible transition at circa 624 °C, which is maintained in the Sc-containing sample (Figure 5c). Irreversible phenomena occur in the pristine alloy above 1150 °C, as in the previous sample. The scandium-containing alloy presents two irreversible endothermic peaks (at 906 and 966 °C) upon heating and two exothermic peaks upon cooling (at 1102 and 1206 °C), which are too far from the heating ones to be considered part of the same phenomenon.

**Figure 6:**
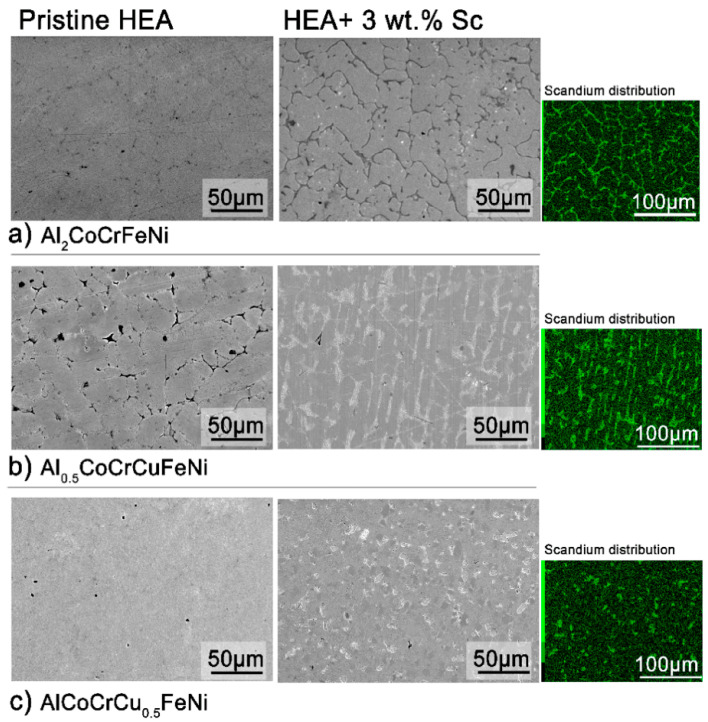
SEM images of the microstructure of annealed Al_2_CoCrFeNi (**a**), Al_0.5_CoCrCuFeNi (**b**) and AlCoCrCu_0.5_FeNi (**c**) before and after 3 wt.% Sc addition. Blank alloys were annealed at 850 °C for 12h; Al_2_CoCrFeNi + 3 wt.% Sc at 900 °C, 12 h; Al_0.5_CoCrCuFeNi and AlCoCrCu_0.5_FeNi at 930 °C, 6h. EDX maps show the scandium element distribution of the areas depicted on the right side (green).

Reversible phenomena thus occur in all systems above 600 °C; whereas irreversible peaks appear in the scandium-containing alloys at ca. 900 °C. To investigate the nature of these transitions, the pristine alloys were annealed above their average first reversible transition temperature, and the scandium-containing specimens at the temperatures of their first irreversible transition. Annealing time was shortened for the samples at the highest temperature (930 °C) in order not to lose aluminum. Therefore, Al_2_CoCrFeNi, Al_0.5_CoCrCuFeNi, and AlCoCrCu_0.5_FeNi were annealed at 850 °C for 12 h. Al_2_CoCrFeNi + 3 wt.% Sc was treated at 900 °C for 12 h, while Al_0.5_CoCrCuFeNi + 3 wt.% Sc and AlCoCrCu_0.5_FeNi + 3 wt.% Sc at 930 °C for 6 h. The corresponding element distribution and microstructures are presented in the following pages. Figure 6 reports the microstructure and element distribution of the annealed Al_2_CoCrFeNi + 3 wt.% Sc alloy. With respect to Figure S1b (the annealed pristine alloy), microstructure is refined and the intermetallic scandium phase grows in a dendritic-like structure. The secondary phase which that have appeared in the pristine alloy (in the form of black dots) is not displayed by the matrix. A comparison with the scandium-containing alloy prior to annealing (Figure S4) shows a more homogeneous distribution of chromium, even though the metal still visibly segregates along the grain boundaries.

The heat treatment of the pristine Al_0.5_CoCrCuFeNi (Figure 6b) causes the disruption of the original dendritic- and interdentritic-like microstructure. However, the microstructure of the scandium-containing alloy after annealing is quite similar to the as-cast pristine alloy: a columnar cellular structure displaying strong element segregation (Figure S2b). The light phase consists mostly of Cu and Sc, and within it regions rich in Al and Ni. The darker areas are rich in Co, Cr, and Fe. It is of interest to note that this element segregation is different from the one prior annealing (Figure S5), as Ni is depleted from the matrix.

The annealed AlCoCrCu_0.5_FeNi + 3 wt.% Sc has a complex microstructure (Figure 6c). Acicular particles grow in an unstructured matrix, the bigger of those being easily removed from the specimen during polishing (and leaving behind elongated valleys). Elemental segregation occurs to form several diverse regions: A Co-rich matrix, areas rich in Al and Ni, and Cu-Sc precipitates. Iron clearly segregates from Al and Ni, but does not follow the trend of other elements. Finally, Cr coalesces in circular well-shaped areas. Combining this information, we can note that the brittle phase is, as expected, rich in Cu and Sc. The annealed scandium-containing alloy is very different not only from the pristine alloy, but also from the original as cast specimen. Indeed, the annealed pristine alloy shows no clustering of Cr, Fe, and Cu (Figure S3); and the scandium-containing as cast alloy clearly highlights the coexistence of Co, Cr, and Fe (Figure S6).

To further investigate the nature of the transitions occurring in our HEA systems and how they are affected by the intermetallic phases, we performed in situ PXRD of disordered CsCl-type Al_2_CoCrFeNi and the *fcc*-structured Al_0.5_CoCrFeNi with and without 3 wt.% scandium. Quantitative data are available only for the main phases, whose reflections had enough intensity to be detected and refined. The development of scandium-rich phases could not be followed directly.

**Figure 7:**
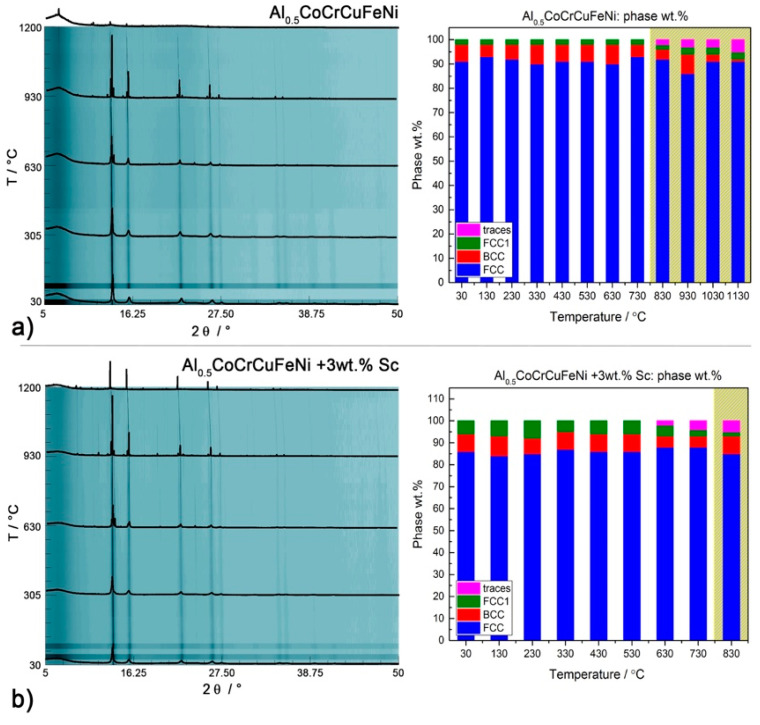
**Left**: High-temperature behavior (I11 at DIAMOND, λ = 0.494984 Å) of Al_0.5_CoCrCuFeNi with (**b**) or without (**a**) scandium according to in situ PXRD data. **Right**: Weight percentage of the major phases in each sample: *fcc* (blue), *bcc* (red), *fcc*_1_ (green), others (pink). The yellow areas mark the start of oxidation.

Several works have highlighted that Al_0.5_CoCrCuFeNi consists of a mixture of two *fcc* phases of similar cell parameters, corresponding to dendritic and interdendritic phase [44,45,46]. Chen reported a single *fcc* structure below 500 °C, and a duplex *fcc/bcc* structure above 600 °C [47]. According to Jones, on the other hand, two *fcc* phases of similar cell parameters co-exist and are stable up until their melting temperatures (1150 °C for the Cu based solid solution and 1350 °C for the multi-component phase) [44]. As shown in Figure 1b, our system consists of an *fcc* phase (*a_fcc_* = 3.601(1) Å) and a small (<10%) amount of *bcc* phase (*a_bcc_* = 2.867(4)). The second *fcc_2_* phase ($a_{fcc_{2}}$ = 3.609(2) Å) reported in Figure 7a can be considered a minor admixture in an exsolution equilibrium with the *bcc* phase. Heating does not affect the primary phase: reflections become sharper, but their relative intensity remains similar. The addition of scandium (Figure 7b) has no impact on the *fcc* matrix, but influences the *bcc* → *fcc*_2_ equilibrium by shifting it towards the formation of *bcc*.

**Figure 8:**
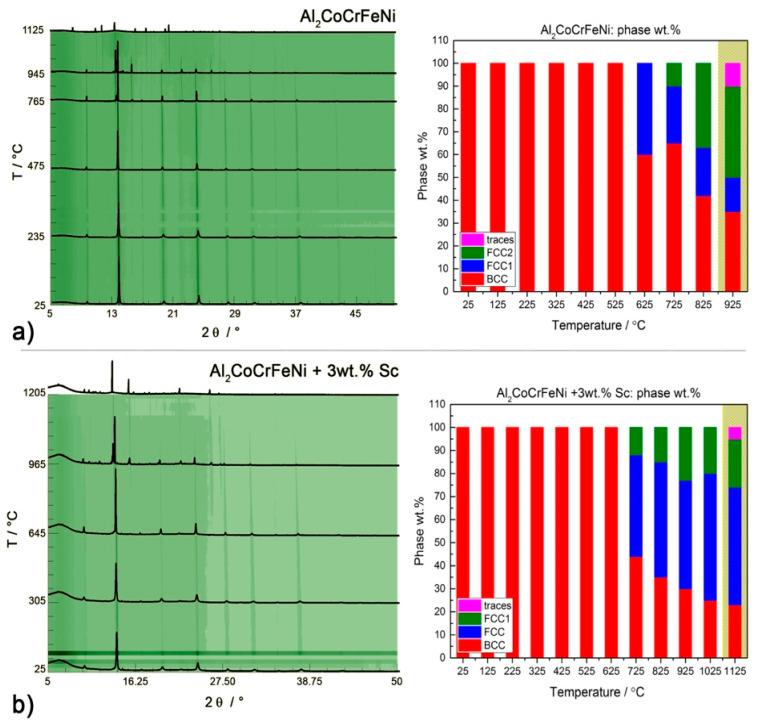
**Left**: High-temperature behavior (I11 at DIAMOND, λ = 0.494984 Å) of CsCl-type Al_2_CoCrFeNi with (**b**) or without (**a**) scandium according to in situ PXRD data. **Right**: Weight percentage of the major phases in each sample: *fcc* (blue), *bcc* (red), *fcc*_1_ (green), others (pink). The yellow areas mark the start of oxidation.

The thermal behavior of the CsCl-type Al_2_CoCrFeNi sample is displayed in Figure 8a. We observe the decomposition of 40 wt.% of the CsCl-type phase above 620 °C and the exsolution of an *fcc* phase ($a_{fcc_{1}}$ = 3.635(8) Å). At 750 °C a second *fcc*_2_ phase of lattice parameter $a_{fcc_{2}}$ = 3.631(9) Å develops from *fcc*_1_ and grows almost linearly with temperature. The continuous presence of the (100) diffraction line in the PXRD pattern of the alloy suggests that the Al-rich sub-structure responsible for the CsCl-type crystal structure might not be involved in the exsolution process. Results for the Sc-containing Al_2_CoCrFeNi alloy are presented in Figure 8b. The exsolution of the *fcc* phase from the CsCl-type one follows a different pathway, with *fcc*_1_ and *fcc*_2_ forming at the same time. The transformation occurs at higher temperatures: a 3 wt.% scandium addition is enough to stabilize the main CsCl-type phase for about 150 °C.

**Figure 9:**
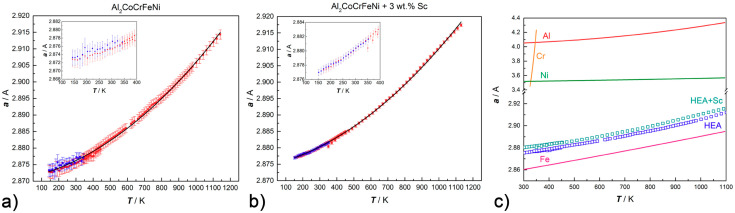
Thermal expansion data fitted with Equation (1) for (**a**) Al_2_CoCrFeNi and (**b**) Al_2_CoCrFeNi + 3 wt.% Sc. In red*:* data collected upon heating from 100–400 K (PETRAIII, λ = 0.207150 Å) and from 300–1100 K (DIAMOND, λ = 0.494984 Å); in blue: data collected upon cooling from 300–100 K (PETRAIII, λ = 0.207150 Å). Low temperature data are highlighted in the inset. (**c**) Thermal expansion curves for Al_2_CoCrFeNi and Al_2_CoCrFeNi + 3 wt.% Sc with respect to their constitutive cubic metals [48,49,50,51].

The thermal expansion coefficient of Al_2_CoCrFeNi and Al_2_CoCrFeNi + 3 wt.% Sc can be evaluated by in situ high-temperature PXRD measurements by fitting the corresponding dataset to:

(1) $$\alpha \left(T\right)=\alpha_{T_{o}}\cdot \exp\left[\alpha \cdot \left(T-T_{o}\right)+\frac{\beta }{2}\cdot \left(T^{2}-T_{o}^{2}\right)\right]$$

where $\alpha_{T_{o}}$ is the cell parameter α at the reference temperature (T_o_). Figure 9 reports the variation of the lattice parameter $\alpha_{\mathrm{CsCl}}$ upon heating and cooling. A satisfactory fitting can be obtained for Al_2_CoCrFeNi for α and β equalling 3.6(2) × 10^−6^·K^−1^ and 1.69(3) × 10^−8^·K^−2^, respectively (Figure 9a); and for Al_2_CoCrFeNi + 3 wt.% Sc for α and β equaling 4.2(1) × 10^−6^× K^−1^ and 1.62(2) × 10^−8^ ×K^−2^, respectively (Figure 9b). As highlighted in Figure 9c, the scandium-containing CsCl-type phase has slightly larger cell parameters than the regular alloy and follows the same trend with temperature. This trend is consistent to the one of pure iron in the investigated temperature range, but differs strongly from the behavior of other cubic metals which constitute the alloy (in particular, from chromium and nickel) [48,49,50,51].

The addition of scandium to the Al-containing HEAs results in the precipitation of a ternary intermetallic of MgZn_2_-type along the grain boundaries. Microstructural data as well as high-temperature PXRD suggest an extraordinary stability of Sc-based precipitates, which form in all alloys containing aluminum and first raw transition metals. The matrix coherence following the formation of the secondary phase is maintained and the Vickers hardness increases up to 20% due to precipitation hardening (for 0.5 wt.% Sc addition to Al_2_CoCrFeNi). The intermetallic phase has high thermal stability and affects the *bcc* → *fcc* exsolution equilibrium by stabilizing the body-centered cubic phase with respect to the face-centered cubic one. This effect is likely related to the segregation of part of the *fcc*-stabilizing elements (i.e., Ni and Co) in the ternary compound. Out of *bcc*-stabilizers, aluminum segregates in the MgZn_2_-type phase, but chromium has much lower affinity for the secondary phase and coalesces in the matrix (as highlighted by EDX maps). Lattice thermal expansion data of the CsCl-type alloy with or without scandium show that both systems follow the same trend upon heating. However, the cubic phase in the scandium-containing HEA has slightly larger cell parameters: this is a further indication of the compositional difference between the matrix of the two systems.

**Figure 10:**
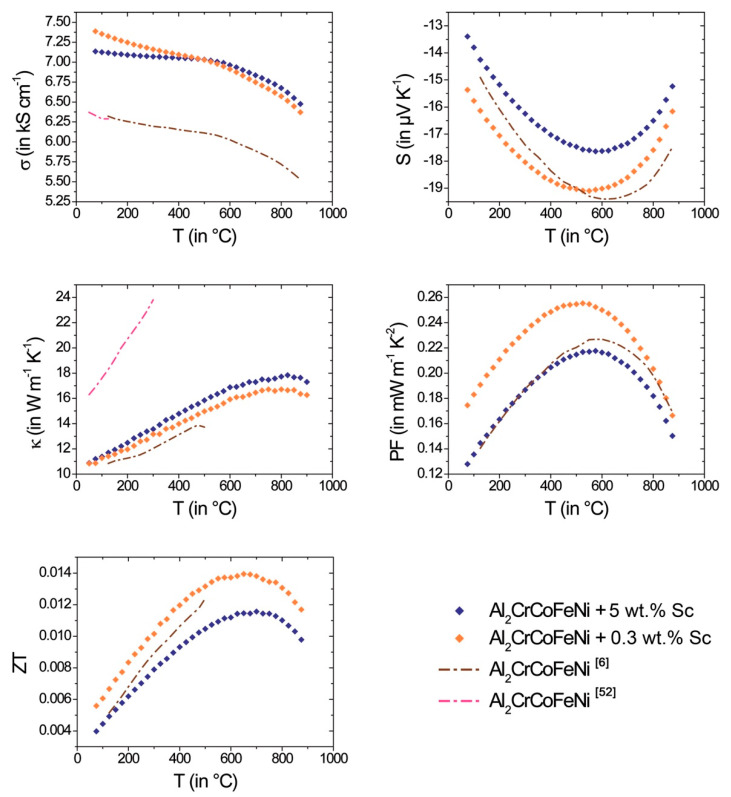
Thermoelectric properties of Al_2_CrCoFeNi + 5 wt.% Sc (blue) and Al_2_CrCoFeNi + 0.3 wt.% Sc (orange) averaged over heating and cooling cycles (without first heating): electrical conductivity σ (**top**, **left**), Seebeck coefficient S (**top**, **right**), thermal conductivity κ (**middle**, **left**), power factor PF (**middle**, **right**) and thermoelectric figure of merit *ZT* (**bottom**, **left**). For comparison, literature data for Al_2_CrCoFeNi (brown [6] and pink [52] dashed line) were added.

While the thermoelectric properties of Al_x_CoCrFeNi (0 ≤ *x* ≤ 3) [6,30,52] are discussed in detail in literature, the effect of Sc alloying on the transport properties of Al_2_CoCrFeNi is herein reported for the first time (Figure 10). The absolute Seebeck coefficient of *n*-type materials decreases slightly with increasing Sc content. The electrical conductivity for both compositions increases by about 1000 S·cm^−1^ compared to pure Al_2_CoCrFeNi [6,52]. In accordance with the higher electrical conductivity, the thermal conductivity shows similar, also slightly increased values compared to ref. [6]; whereas the thermal conductivity for pure Al_2_CrCoFeNi from ref. [52] is extraordinarily high.

Power factor and *ZT* value increase with temperature until a peak *ZT* value of 0.012 at 700 °C (for 5 wt.% Sc addition) and 0.014 at 650 °C (for 0.3 wt.% Sc) is reached. The subsequent decrease of *ZT* with temperature is mainly attributed to the decrease of the absolute value of *S* due to excitation of minority charge carriers. According to PXRD patterns of the samples after thermoelectric measurements, no degradation was observed (Figure S12). In addition, the samples show a good cyclability within three subsequent heating and cooling cycles (Figures S13 and S14).

## 4. Conclusions

Ever since HEAs have been proposed as promising materials for high-temperature applications, their phase stability, and the effect of selected intermetallic compounds on their performances upon heating have seldom been studied. We report a thorough investigation on the effect of small scandium additions to the microstructure, phase stability, and mechanical and thermoelectric properties of the model HEAs Al_2_CoCrFeNi, Al_0.5_CoCrCuFeNi and AlCoCrCu_0.5_FeNi. High-temperature in situ PXRD investigations highlights the existence of secondary phases in these systems, traditionally though as consisting of a single solid solution. The addition of scandium to the studied HEAs causes the precipitation of a MgZn_2_-type intermetallic phase. This hexagonal compound leads to chemical inhomogeneity along grain boundaries, causing grain refinement and a powerful increase in hardness (for Al_2_CoCrFeNi, a 0.5 wt.% addition of scandium enhances hardness by 20%). Upon heating, *bcc* and CsCl-type phases turn into one or more *fcc* phases, whereas *fcc* phases appears very stable. Scandium acts as stabilizer of the *bcc* phase, by affecting the exsolution equilibrium *bcc* → *fcc*. Regarding thermoelectric properties, the alloying of scandium in Al_2_CoCrFeNi increases the electrical conductivity by 14%. The maximum *ZT* value of 0.014 reached for 0.3 w% Sc addition at 650 °C is in the same order of magnitude as for other HEAs, but too low to make these materials promising for current thermoelectric applications. Still, HEAs are a young material class and optimized HEA will have the advantage of extreme thermal stability.

The results reported here open new possibilities in the design and further improvement of the properties of multicomponent alloys. They could be used as the basis for designing new stable and metastable multicomponent single-phase alloys with improved mechanical properties. The effect of minor elements additions to HEA should be thoroughly investigated to tackle specific technological needs.

## Supplementary Materials

The following are available online at http://www.mdpi.com/1099-4300/20/7/488/s1.

## References

[1] Miracle D.B., Senkov O.N. (2017). A critical review of high entropy alloy and related concepts. Acta Mater..

[2] Zhang W., Liaw P.K., Zhang Y. (2018). Science and technology in high-entropy alloys. Sci. China Mater..

[3] Dahlborg U., Cornide J., Calvo-Dahlborg M., Hansen T.C., Fitch A., Leong Z., Chambreland S., Goodall R. (2016). Structure of some CoCrFeNi and CoCrFeNiPd multicomponent HEA alloys by diffraction techniques. J. Alloy Compd..

[4] Yeh J.W., Chen S.K., Lin S.J., Gan J.Y., Chin T.S., Shun T.T., Tsau C.H., Chang S.Y. (2004). Nanostructured high-entropy alloys with multiple principal elements: novel alloy design concepts and outcomes. Adv. Eng. Mater..

[5] Cantor B., Chang I.T.H., Knight P., Vincent A.J.B. (2004). Microstructural development in equiatomic multicomponent alloys. Mater. Sci. Eng. A.

[6] Shafeie S., Guo S., Hu Q., Fahlquist H., Erhart P., Palmqvist A. (2015). High-entropy alloys as high-temperature thermoelectric materials. J. Appl. Phys..

[7] Samaei A.T., Mirsayar M.M., Aliha M.R.M. (2015). The microstructure and mechanical behavior of modern high temperature alloys. Eng. Solid Mech..

[8] Antonaglia J., Xie X., Tang Z., Tsai. C.-W., Qiao J.W., Zhang Y., Laktionova M.O., Tabachnikova E.D., Yeh J.W., Senkov O.N. (2014). Temperature effect on deformation and serration behavior of high-entropy alloys (HEAs). JOM.

[9] Manzoni A.M., Singh S., Daoud H.M., Popp R., Völkl R., Glatzel U., Wanderka N. (2016). On the path to optimizing the Al-Co-Cr-Co-Fe-Ni-Ti high entropy alloy family for high temperature applications. Entropy.

[10] Santodonato L.J., Zhang Y., Feygenson M., Parish C.M., Gao M.C., Weber R.J.W., Neuefeind J.C., Tang Z., Liaw P.K. (2015). Deviation from high-entropy configurations in the atomic distributions of a multi-principal-element alloy. Nat. Comm..

[11] Yusenko K.V., Riva S., Crichton W.A., Spektor K., Bykova E., Pakhomova A., Tudball A., Kupenko I., Rohrbach A., Klemme S. (2018). High-pressure high-temperature tailoring of High-Entropy Alloys for extreme environments. J. Alloy Compd..

[12] Prasad H., Singh S., Panigrahi B.B. (2017). Mechanical activated synthesis of alumina dispersed FeNiCoCrAlMn high entropy alloy. J. Alloy Compd..

[13] Rogal L., Kalita D., Tarasek A., Bobrowski P., Czerwinski F. (2017). Effect of SiC nano-particles on microstructure and mechanical properties of the CoCrFeMnNi high entropy alloy. J. Alloy Compd..

[14] Riva S., Tudball A., Mehraban S., Lavery N.P., Brown S.G.R., Yusenko K.V. (2018). A novel high-entropy alloy-based composite material. J. Alloy Compd..

[15] Hsu U.S., Hung U.D., Yeh J.W., Chen S.K., Huang Y.S., Yang C.C. (2007). Alloying behavior of iron, gold and silver in AlCoCrCuNi-based equimolar high-entropy alloys. Mater. Sci. Eng. A.

[16] Zhu J.M., Fu H.M., Zhang H.F., Wang A.M., Li H., Hu Z.Q. (2010). Synthesis and properties of multiprincipal component AlCoCrFeNiSi_x_ alloys. Mater. Sci. Eng. A.

[17] Li B.S., Wang Y.P., Ren M.X., Yang C., Fu H.Z. (2008). Effects of Mn, Ti and V on the microstructure and properties of AlCrFeCoNiCu high entropy alloys. Mater. Sci. Eng. A.

[18] Riva S., Yusenko K.V., Lavery N.P., Jarvis D.J., Brown S.G.R. (2016). The scandium effect in multicomponent alloys. Int. Mater. Rev..

[19] Riva S., Fung C.M., Searle J.R., Clark R.N., Lavery P.N., Brown S.G.R., Yusenko K.V. (2016). Formation and disruption of W-phase in High-Entropy Alloys. Metals.

[20] Tung C.-C., Yeh J.-W., Shun. T.-T., Chen S.-K., Huang Y.-S., Chen H.-C. (2007). On the elemental effect of AlCoCrCuFeNi high-entropy alloy system. Mater. Lett..

[21] Li C., Li J.C., Zhao M., Jiang Q. (2010). Effect of aluminum contents on microstructure and properties of AlxCoCrFeNi alloys. J. Alloy Compd..

[22] Kao Y.F., Chen T.J., Chen S.K., Yeh J.W. (2009). Microstructure and mechanical property of as-cast, -homogenized and -deformed AlxCoCrFeNi (0 ≤ x ≤2) high-entropy alloys. J. Alloy Compd..

[23] Li B.-Y., Peng K., Hu A.-P., Zhou L.-P., Zhu J.-J., Li D.-Y. (2013). Structure and properties of FeCoNiCrCo0.5Alx high-entropy alloys. Trans. Nonferrous Met. Soc. China.

[24] Norris S.D., Parker J.D. (1996). Deformation processes during disc bend loading. Mater. Sci. Technol..

[25] Thompson S.P., Parker J.E., Marchal J., Potter J., Birt A., Yuan F., Fearn R.D., Lennie A.R., Street S.R., Tang C.C. (2011). Fast X-ray powder diffraction on I11 at Diamond. J. Synchrotron Radiat..

[26] Rajiv P., Dinnebier R., Jansen M. (2010). Powder 3D Parametric: A program for automated sequential and parametric Rietveld refinement using Topas. Mater. Sci. Forum.

[27] Kabekkodu S. (2012). PDF-2 Release 2012 (Database).

[28] TOPAS v.4.0, Bruker-AXS 5465 East Cheryl Parkway–Bruker AXS–2009. https://www.bruker.com/products/x-ray-diffraction-and-elemental-analysis/x-ray-diffraction/xrd-software/topas.html.

[29] Dusza L. (1995). Combined Solution of the Simultaneous Heat Loss and Finite Pulse Corrections with the Laser Flash Method. High Temp.–High Press..

[30] Uporov S., Bykov V., Pryanichnikov S., Shubin A., Uporova N. (2017). Effect of synthesis route on structure and properties of AlCoCrFeNi high-entropy alloy. Intermetallics.

[31] Ye Y.F., Wang Q., Zhao Y.L., He Q.F., Lu J., Yang Y. (2016). Elemental segregation in solid-solution high-entropy alloys: Experiments and modelling. J. Alloy Compd..

[32] Calvo-Dahlborg M., Brown S.G.R. (2017). Hume-Rothery for HEA classification and self-organizing map for phases and properties prediction. J. Alloy Compd..

[33] Lucas M.S., Mauger L., Munoz J.A., Xiao Y.M., Sheets A.O., Semiatin S.L., Horwath J., Turgut Z. (2011). Magnetic and vibrational properties of high-entropy alloys. J. Appl. Phys..

[34] Li C., Zhao M., Li J.C., Jiang Q. (2008). B2 structure of high-entropy alloys with addition of Al. J. Appl. Phys..

[35] Tang Z., Yuan T., Tsai C.-W., Yeh J.-W., Lundin C.D., Liaw P.K. (2015). Fatigue behavior of a wrought Al0.5CoCrCuFeNi two-phase high-entropy alloy. Acta Mater..

[36] Novak T.G., Vora H.D., Mishra R.S., Young M.L., Dahotre N.B. (2014). Synthesis of Al_0.5_CoCrCuFeNi and Al_0.5_CoCrFeMnNi high-entropy alloys by laser melting. Metall. Mater. Trans. B.

[37] Wang W.-R., Wang W.-L., Wang S.-C., Tsai Y.-C., Lai C.-H., Yeh J.-W. (2012). Effects of Al addition on the microstructure and mechanical property of Al_x_CoCrFeNi high-entropy alloys. Intermetallics.

[38] Sudavtsova V.S., Shevchenko M.O., Berezutskii V.V., Ivanov M.I. (2013). Thermodynamic properties of liquid Fe-Sc alloys. Powder Metall. Met. Ceram..

[39] Shevchenko M.A., Ivanov M.I., Berezutskii V.V., Kudin V.G., Sudavtsova V.S. (2014). Thermodynamic properties of alloys in the Ni-Sc and Ni-Y systems. Russ. J. Phys. Chem. A.

[40] Shevchenko M.A., Berezutskii V.V., Ivanov M.I., Kudin V.G., Sudatsova V.S. (2014). Thermodynamic properties of the alloys of the Al-Co and Al-Co-Sc systems. Russ. J. Phys. Chem. A.

[41] Gao M.C., Yeh J.-W., Liaw P.K., Zhang Y. (2016). High-Entropy Alloys: Fundamentals and applications.

[42] Jones N.G., Frezza A., Stone H.J. (2014). Phase equilibria in an Al_0.5_CrFeCoNiCu high entropy alloy. Mater. Sci. Eng. A.

[43] Jones N.G., Christofidou K.A., Stone H.J. (2015). Rapid precipitation in an Al_0.5_CrFeCoNiCu high entropy alloy. Mater. Sci. Technol..

[44] Jones N.G., Izzo R., Mignanelli P.M., Christofidou K.A., Stone H.J. (2016). Phase evolution in an Al_0.5_CrFeCoNiCu high entropy alloy. Intermetallics.

[45] Jones N.G., Aveson J.W., Bhowmik A., Conduit B.D., Stone H.J. (2014). On the entropic stabilization of an Al_0.5_CrFeCoNiCu. Intermetallics.

[46] Pickering E.J., Stone H.J., Jones N.G. (2015). Fine-scale precipitation in the high-entropy alloy Al_0.5_CrFeCoNiCu. Mater. Sci. Eng. A.

[47] Chen S., Xie X., Chen B., Qiao J., Zhang Y., Ren Y., Dahmen K.A., Liaw P.K. (2015). Effects of temperature on serrated flows of Al_0.5_CoCrCuFeNi High-Entropy Alloy. JOM.

[48] Figgins B.F., Jones G.O., Riley D.P. (1956). LXXVII. The thermal expansion of aluminium at low temperatures as measured by an X-ray diffraction method. Philos. Mag..

[49] Basinski Z.S., Hume-Rothery W., Sutton A.L. (1955). The lattice expansion of iron. Proc. R. Soc. Lond. A.

[50] Owen E.A., Yates E.L. (1936). LXVI. X-ray measurement of the thermal expansion of pure nickel. Lond. Edinb. Dublin Philos. Mag. J. Sci..

[51] Ross R.G., Hume-Rothery W. (1963). High temperature X-ray metallography: I. A new Debye-Sherrer camera for use at very high temperatures II. A new parafocusing camera III. Applications to the study of chromium, hafnium, molybdenum, rhodium, ruthenium and tungsten. J. Less Common Met..

[52] Chou H.-P., Chang Y.-S., Chen S.-K., Yeh J.-K. (2009). Microstructure, thermophysical and electrical properties in Al_x_CoCrFeNi (0 ≤ x ≤ 2) high-entropy alloys. Mater. Sci. Eng. B.

