# Supplementary material for: The Effect of Scandium Ternary Intergrain Precipitates in Al-Containing High-Entropy Alloys
Source: Entropy (Basel). 2018 Jun 22;20(7):488. doi: 10.3390/e20070488 (PMC7513013; doi:10.3390/e20070488)
Supplement: Supplementary file 1 [file entropy-20-00488-s001.pdf]

SUPPLEMENTARY INFORMATION

The effect of alloying with scandium in Al-containing High-Entropy Alloys

Sephira Riva [1], Shahin Mehraban [1], Nicholas P. Lavery [1], Stefan Schwarzmüller [2], Oliver Oeckler [2], Stephen G. R. Brown [1], and Kirill V. Yusenko [1,3,*]

[1] College of Engineering, Swansea University, Swansea SA1 8EN, Wales, UK
[2] Leipzig University, Faculty of Chemistry and Mineralogy; Institute for Mineralogy, Crystallography and Materials Science; Scharnhorststr. 20, 04275 Leipzig, Germany
[3] Institute of Solid State Chemistry, Pervomaiskaia str. 91, 620990 Ekaterinburg, Russia

Table S1. Rationale of the alloys presented in this work, with the corresponding characterization. The acronyms RT, HT and LT refer to synchrotron experiments performed at room temperature, high-temperature and low-temperature respectively. The notation Thermoelectric properties includes the measurement of Seebeck coefficient, electrical conductivity and thermal diffusivity.

Alloy	Form	Performed analysis
$Al_2CoCrFeNi$	As cast pellet	SEM-EDX, Density, Vickers hardness, DSC, Disc punch test
	Annealed pellet (850 °C, 12h)	SEM-EDX, RT PXRD
	Powder from as-cast pellet	RT PXRD, HT PXRD, LT PXRD
$Al_2CoCrFeNi$ + 0.3wt.% Sc	As-cast pellet	SEM-EDX, RT PXRD, Thermoelectric properties
$Al_2CoCrFeNi$ + 0.5wt.% Sc	As-cast pellet	SEM-EDX, RT PXRD, Disc punch test
$Al_2CoCrFeNi$ + 2wt.% Sc	As-cast pellet	SEM-EDX, RT PXRD, Disc punch test
$Al_2CoCrFeNi$ + 3wt.% Sc	As-cast pellet	SEM-EDX, Density, RT PXRD, Vickers hardness, DSC
	Annealed pellet (900 °C, 12h)	SEM-EDX, RT PXRD
	Powder from as-cast pellet	RT PXRD, HT PXRD, LT PXRD
$Al_2CoCrFeNi$ + 5wt.% Sc	As-cast pellet	SEM-EDX, RT PXRD, Thermoelectric properties
$Al_{0.5}CoCrCuFeNi$	As-cast pellet	SEM-EDX, Vickers hardness, DSC
	Annealed pellet (850 °C. 12h)	SEM-EDX
	Powder from as-cast pellet	RT PXRD, HT PXRD
$Al_{0.5}CoCrCuFeNi$ + 0.5wt.% Sc	As-cast pellet	SEM-EDX, Vickers hardness
$Al_{0.5}CoCrCuFeNi$ + 2wt.% Sc	As-cast pellet	SEM-EDX, Vickers hardness
$Al_{0.5}CoCrCuFeNi$ + 3wt.% Sc	As-cast pellet	SEM-EDX, Vickers hardness, DSC
	Annealed pellet (930 °C, 6h)	SEM-EDX
	Powder from as-cast pellet	RT PXRD, HT PXRD
$AlCoCrCu_{0.5}FeNi$	As-cast pellet	SEM-EDX, Vickers hardness, DSC, Disc punch test
	Annealed pellet (850 °C, 12h)	SEM-EDX
	Powder from as-cast pellet	RT PXRD
$AlCoCrCu_{0.5}FeNi$ + 0.5wt.% Sc	As-cast pellet	SEM-EDX, Vickers hardness
$AlCoCrCu_{0.5}FeNi$ + 2wt.% Sc	As-cast pellet	SEM-EDX, Vickers hardness

$AlCoCrCu_{0.5}FeNi$ + 3wt.% Sc	As-cast pellet	SEM-EDX, Vickers hardness, DSC, Disc punch test
	Annealed pellet (930 °C, 6h)	SEM-EDX
	Powder from as-cast pellet	RT PXRD

Atomic composition and element distribution according to EDX maps.

Table S2. Atomic composition of the synthesized samples according to EDX map (x500, x2000).

	$Al_2CoCrFeNi$ at.%		$Al_2CoCrFeNi$ + 3wt.% Sc		$Al_{0.5}CoCrCuFeNi$ at.%		$Al_{0.5}CoCrCuFeNi$ + 3wt.% Sc		$AlCoCrCu_{0.5}FeNi$ at.%		$AlCoCrCu_{0.5}FeNi$ + 3wt.% Sc	
	As melted (±0.04)	Anneal. (±0.04)	Grain at.% (±0.05)	Inter-grain at.% (±0.03)	As melted (±0.03)	Anneal. (±0.04)	Grain at.% (±0.04)	Inter-grain at.% (±0.04)	As melted (±0.03)	Anneal. (±0.04)	Grain at.% (±0.05)	Inter-grain at.% (±0.04)
Al	44.4(3)	39.5(1)	29.2(6)	18.4(2)	13.2(2)	6.6(3)	7.2(6)	2.8(5)	22.8(2)	27.5(7)	10.7(1)	8.8(3)
Co	14.7(2)	15.7(7)	19.6(4)	16.2(8)	17.9(4)	19.8(3)	20.4(0)	3.2(3)	17.2(5)	16.8(0)	21.3(3)	10.6(0)
Cr	13.6(2)	13.8(2)	14.1(8)	11.1(1)	16.6(6)	18.0(6)	17.3(2)	1.6(5)	17.6(0)	16.0(6)	29.9(3)	6.3(2)
Cu	-	-	-	-	16.9(4)	11.1(9)	16.2(7)	75.7(4)	8.6(7)	6.9(7)	6.0(1)	51.8(6)
Fe	14.3(4)	15.1(4)	16.5(1)	17.7(1)	18.5(1)	20.1(4)	20.2(3)	2.4(8)	17.8(4)	16.8(9)	23.2(5)	8.6(1)
Ni	12.8(9)	15.7(7)	19.9(9)	19.4(5)	17.5(2)	17.6(8)	18.2(6)	10.6(5)	15.8(1)	15.7(1)	14.6(3)	13.0(4)
Sc	-	-	0.4(2)	17.0(2)	-	-	0.2(2)	3.4(1)	-	-	0.1(4)	0.7(5)

Element distribution of the as-cast and annealed alloys according to EDX maps

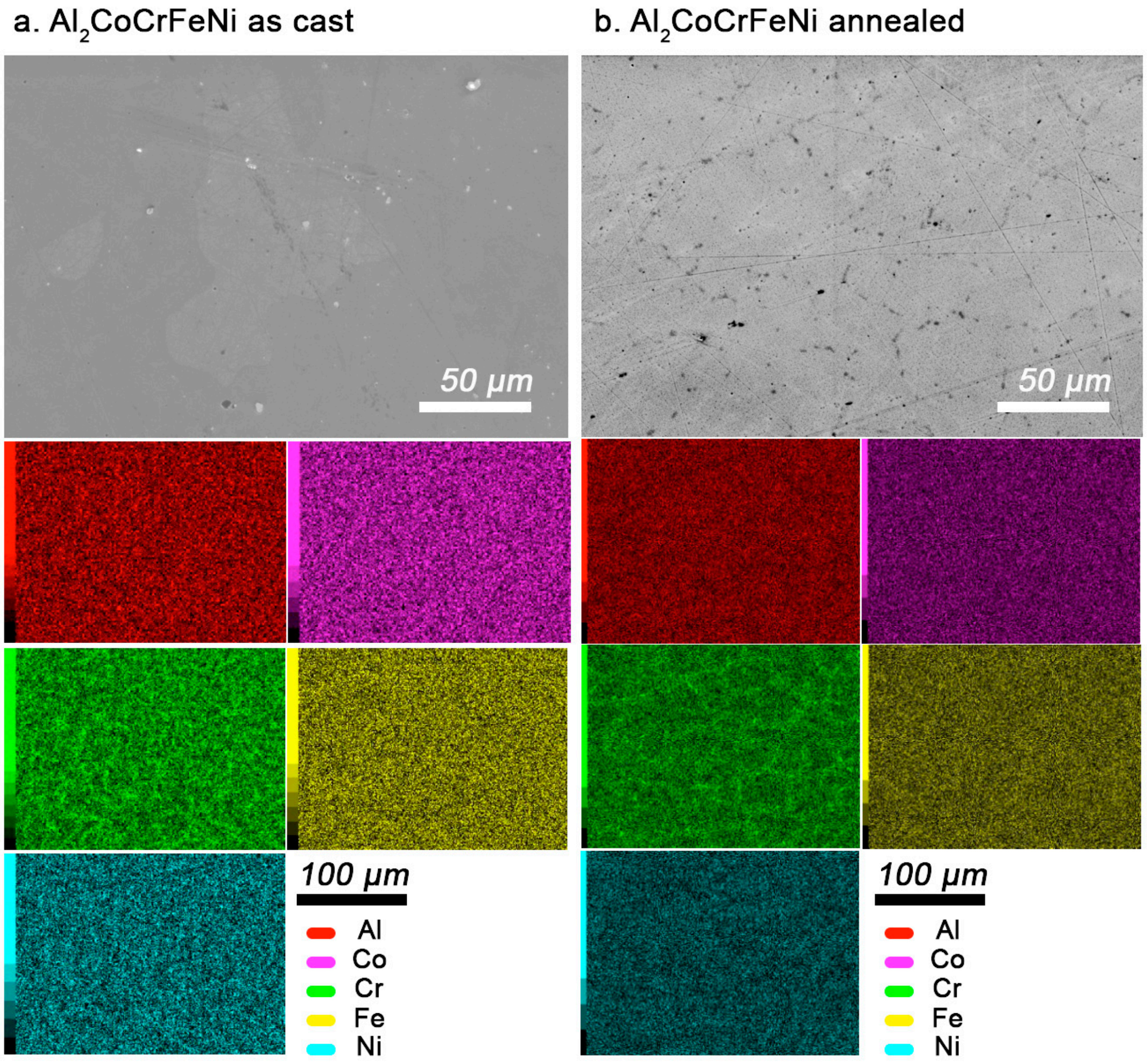

Figure S1. BSE images and element distribution of as cast and annealed $Al_2CoCrFeNi$ samples according to EDX. From top to bottom and left to right, elements are: Al, Co, Cr, Fe, Ni.

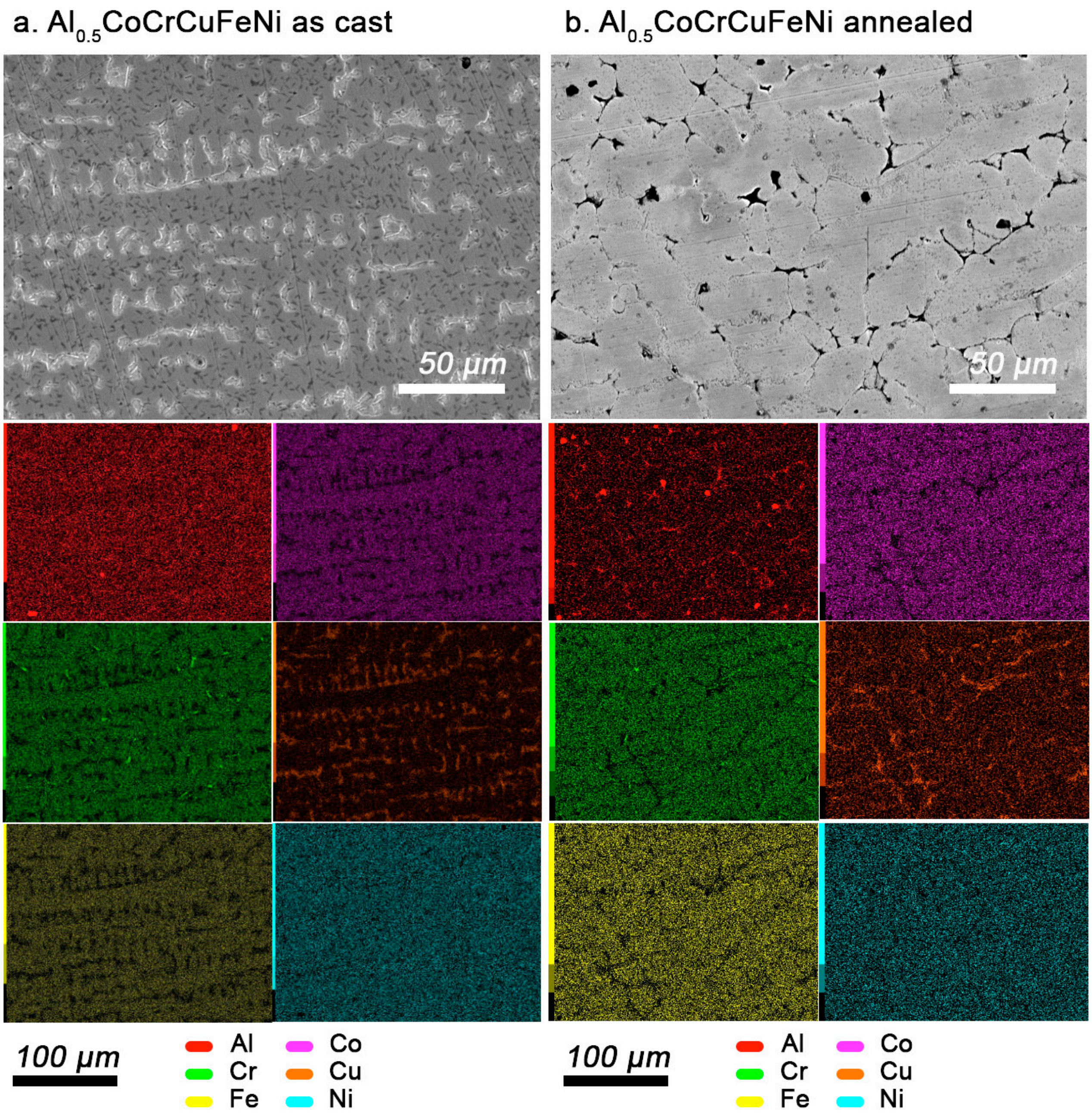

Figure S2. BSE images and element distribution of as cast and annealed $Al_{0.5}CoCrCuFeNi$ samples according to EDX. From top to bottom and left to right, elements are: Al, Co, Cr, Cu, Fe, Ni.

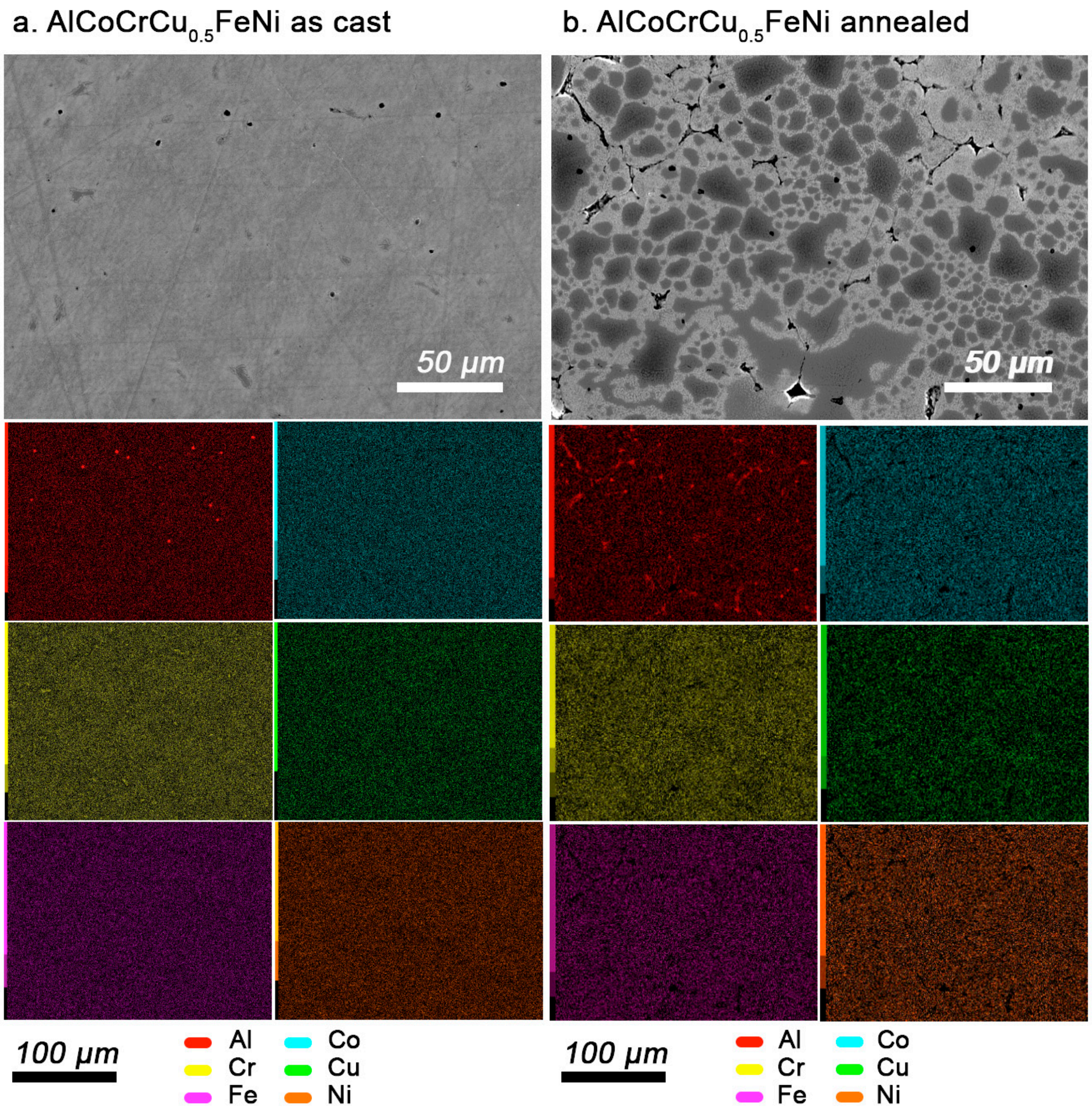

Figure S3. BSE images and element distribution of as cast and annealed $AlCoCrCu_{0.5}FeNi$ samples according to EDX. From top to bottom and left to right, elements are: Al, Co, Cr, Cu, Fe, Ni.

Element distribution of the as-cast Sc-containing alloys according to EDX maps

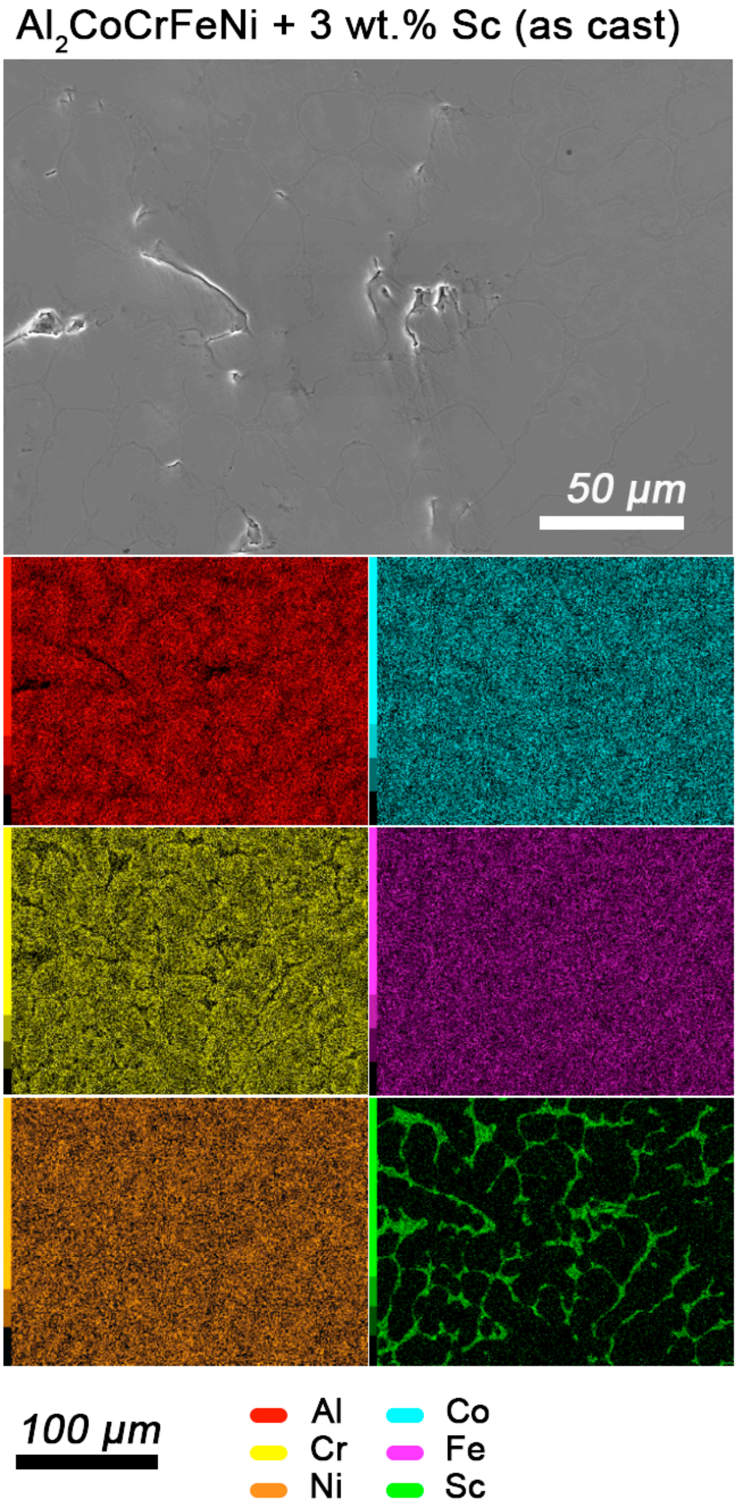

Figure S4. BSE image and element distribution of as cast $Al_2CoCrFeNi$ +3wt.%Sc samples according to EDX. From top to bottom and left to right, elements are: Al, Co, Cr, Fe, Ni and Sc.

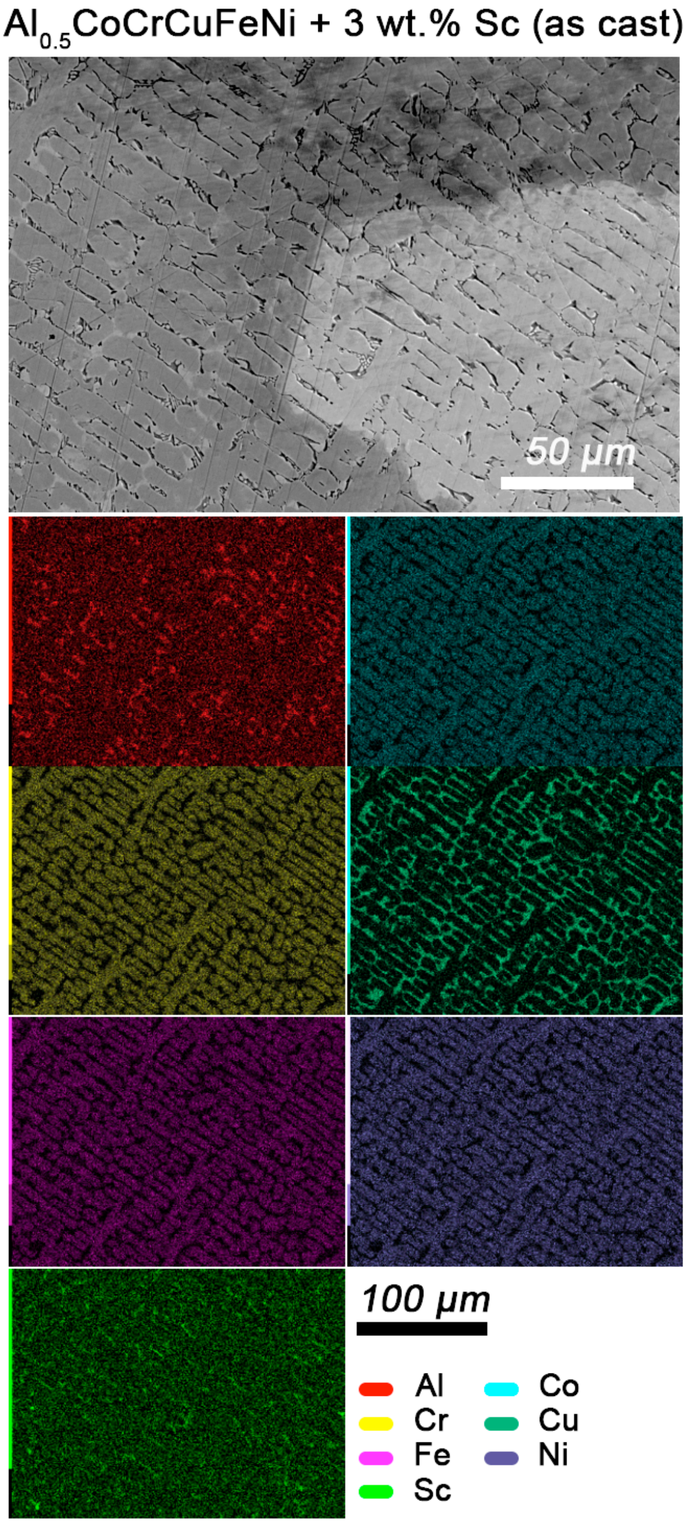

Figure S5. BSE image and element distribution of as cast $Al_{0.5}CoCrCuFeNi$ +3wt.%Sc samples according to EDX. From top to bottom and left to right, elements are: Al, Co, Cr, Cu, Fe, Ni and Sc.

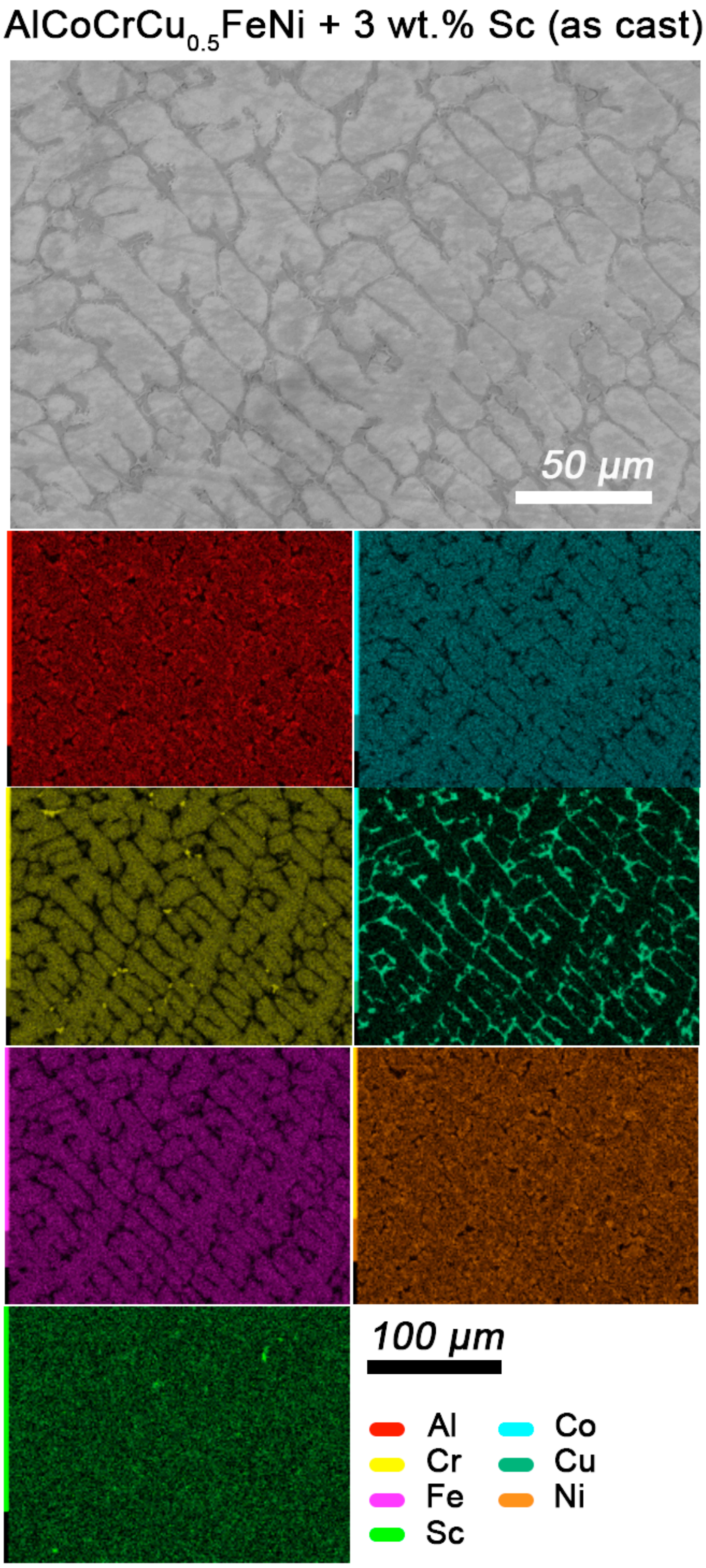

Figure S6. BSE image and element distribution of as cast $AlCoCrCu_{0.5}FeNi$ +3wt.%Sc samples according to EDX. From top to bottom and left to right, elements are: Al, Co, Cr, Cu, Fe, Ni and Sc.

Mechanical properties: disk-punch test

Applied force was plotted against disk's displacement. To normalize the disks for the standard 0.5 mm thickness, the following equation(s) were used:
Ultimate tensile strength for brittle materials [MPa = Nmm^{-2}] [1]

$$Cl = A - (D + 2t) = 8 - (4 + 2t)$$

$$UTS = \frac{F_m}{t(0.14D - 0.82Cl + 2.17u_m + 0.6)}$$

Where F_m is the maximum load during PT, *t* is the disk thickness in mm, *D* is the punch diameter (4 mm), *Cl* is the die clearance in mm and u_m is the displacement at failure. In the first equation, *A* is the diameter of the lower die (8 mm).
Punch test results obtained after normalizing thickness to 0.5mm: [2]

The inflexion point is a constant in the following equations.

For $P_{test} < P_{inflexion}$

$$P_{0.5} = 0.5^2\left(\frac{P_{test}}{t^2}\right)$$

For $P_{test} > P_{inflexion}$

$$P_{0.5} = 0.5\left(\frac{P_{test}}{t}\right) + 0.5\,P_{inflexion}\left(\frac{0.5 - t}{t^2}\right)$$

Ultimate tensile strength [MPa = Nmm^{-2}]

$$UTS = \frac{0.4964\,F_m}{u_m h_o} - 94.146$$

Yield stress

$$YS = \frac{0.4454\,F_e}{h_o^2} + 86.866$$

Where F_m is the maximum load during PT, u_m is the displacement related to the maximum load F_m, h_o is the normalized thickness (0.5mm) and F_e is the load illustrating the conversion between linearity and yield zone (intersection between zone 1 and 2). [3]
Fracture stress for brittle materials

$$\sigma_f = 130\frac{F_m}{t^2} - 320$$

Fracture toughness for brittle materials

$$K_{IC} = 0.07(\,\sigma_f)^{2/3}$$

The units of KIC are MPa, F_m is in N and *t* is the initial thickness in mm. [4]

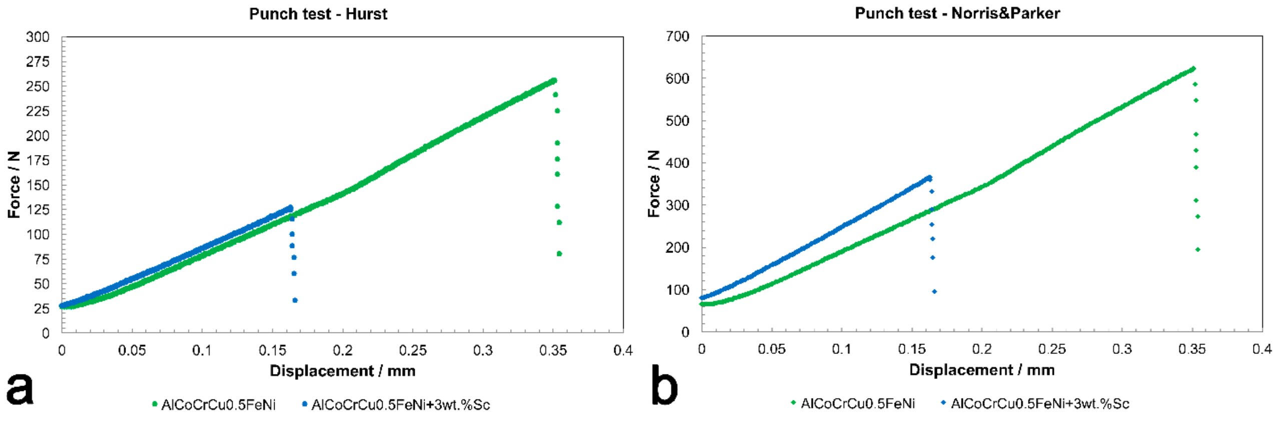

Figure S7. Disk punch test for the $AlCoCrCu_{0.5}FeNi$ HEA with 0 and 3wt.% Sc, data elaborated according to the equations reported by Norris and Parker (*right*) and Hurst (*left*) [1], [3].

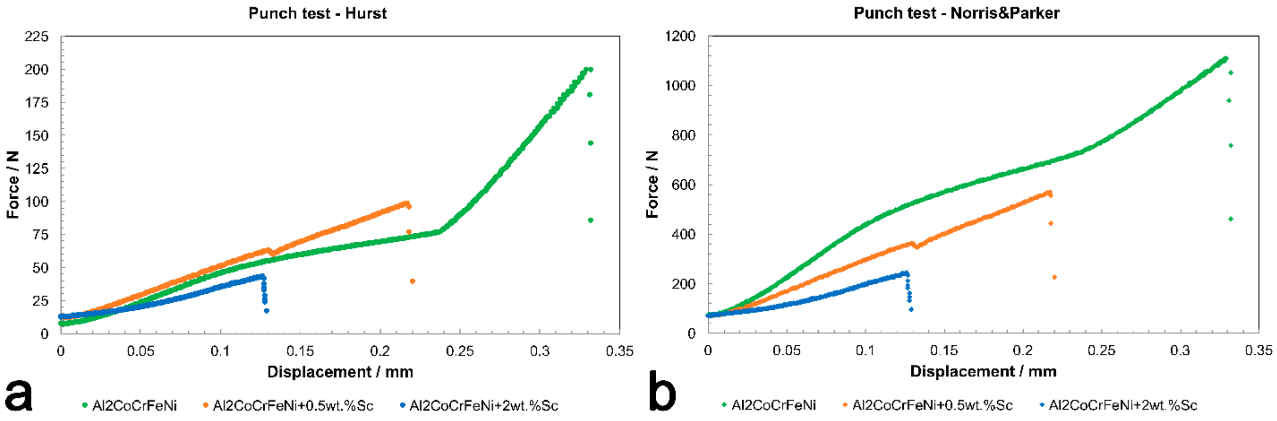

Figure S8. Disk punch test for the $Al_2CoCrFeNi$ alloy with 0, 0.5 and 2wt.% Sc, data elaborated according to the equations reported by Hurst (*left*) and Norris and Parker (*right*) [1], [3].

Element distribution of the annealed alloys according to EDX maps

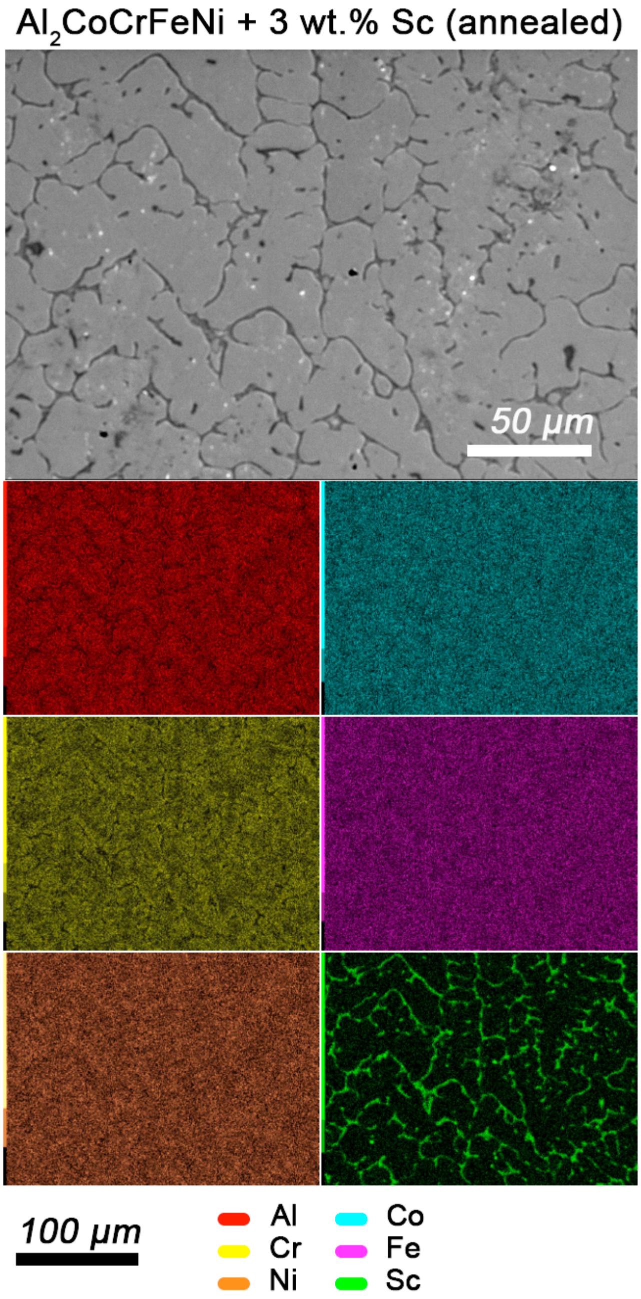

Figure S9. BSE image and element distribution of $Al_2CoCrFeNi$+3wt.%Sc samples after annealing at 900°C, 12h. Data from EDX maps. From top to bottom and left to right, elements are: Al, Co, Cr, Fe, Ni and Sc.

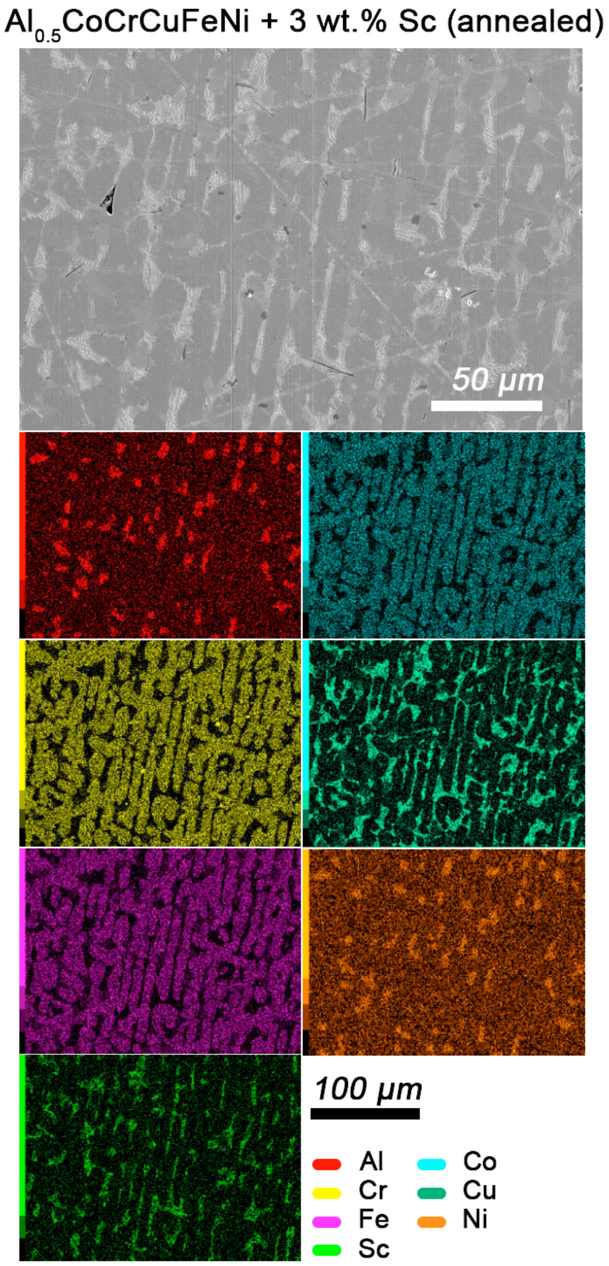

Figure S10. BSE image and element distribution in $Al_{0.5}CoCrCuFeNi$ +3wt.%Sc samples after annealing at 930°C, 6h, respectively. Data from EDX maps. From top to bottom and left to right, elements are: Al, Co, Cr, Cu, Fe, Ni and Sc.

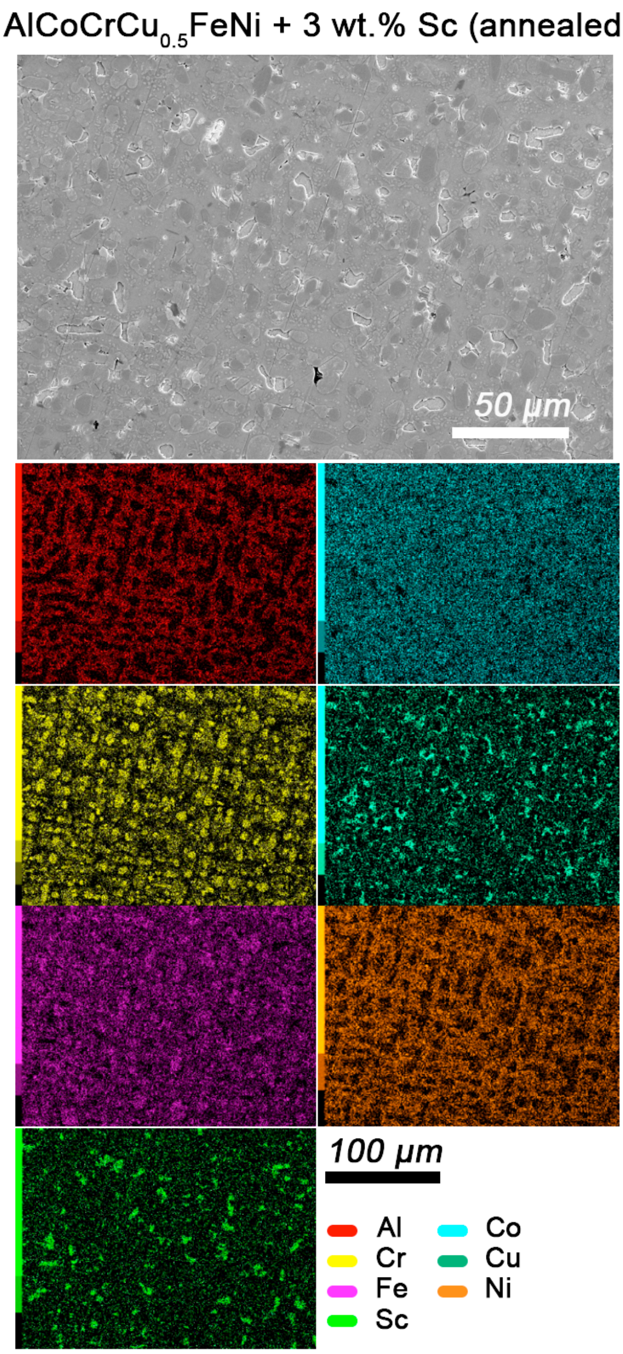

Figure S11. BSE image and element distribution of $AlCoCrCu_{0.5}FeNi$ +3wt.%Sc samples after annealing at 930°C, 6h, respectively. Data from EDX maps. From top to bottom and left to right, elements are: Al, Co, Cr, Cu, Fe, Ni and Sc.

Electrical and thermal transport measurements

Table S3. Density and Dulong-Petit heat capacity used for the calculation of thermal conductivity.

**Composition**	**Density (in g cm^{-3})**	**C_p (Dulong-Petit, in J g^{-1} K^{-1})**
$Al_2CoCrFeNi$ + 5 w% Sc	5.99	0.53651
$Al_2CoCrFeNi$ + 0.3 w% Sc	6.35	0.53565

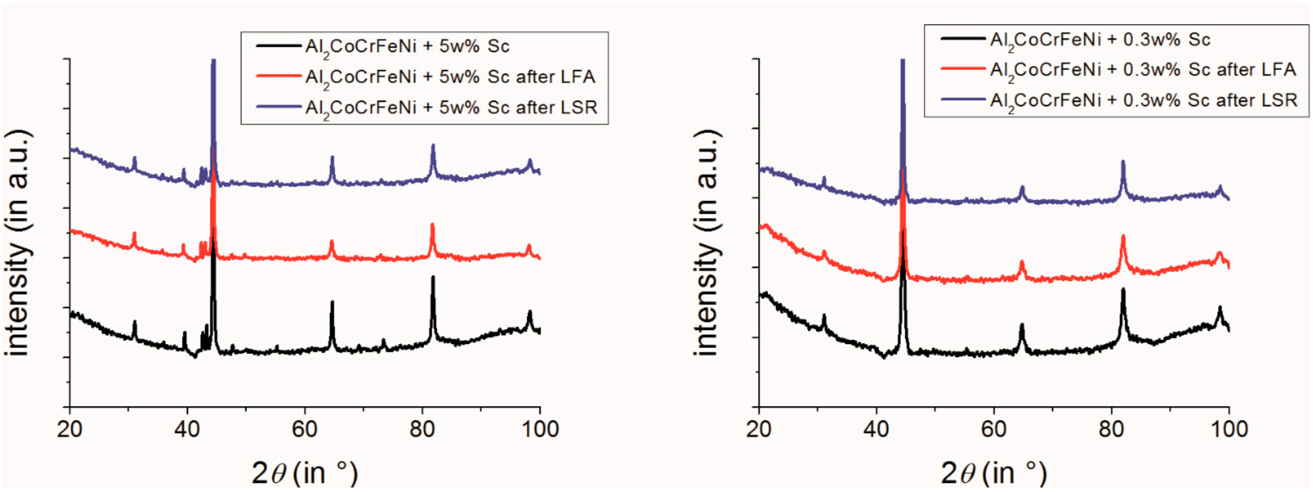

Figure S12. PXRD data of Al2CoCrFeNi + 5 w% Sc (left) and Al2CoCrFeNi + 0.3 w% Sc (right) before thermoelectric measurements (black) and after LFA (red) and LSR (blue), respectively.

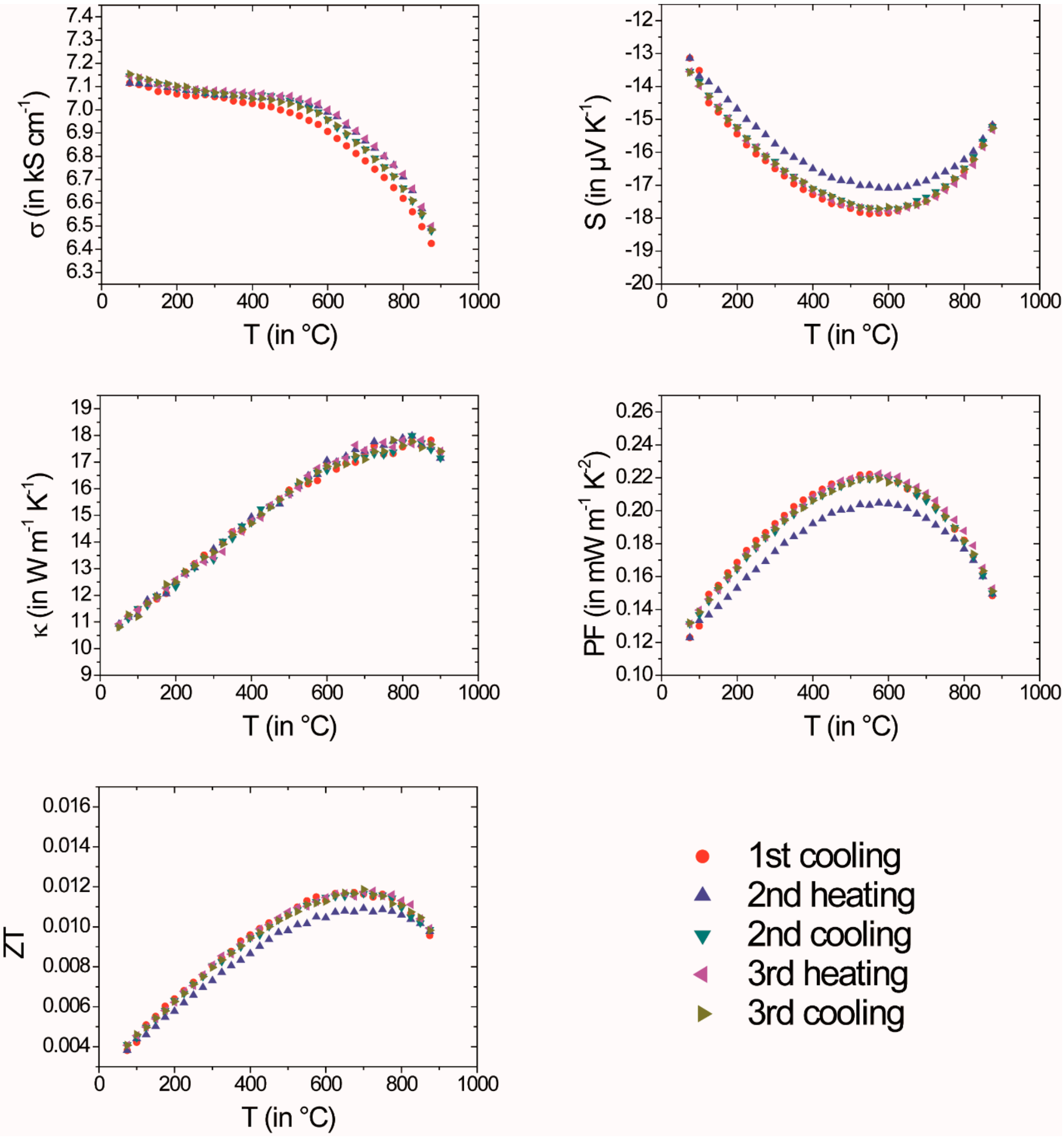

Figure S13. Thermoelectric properties of $Al_2CrCoFeNi$ + 5 w% Sc for three consecutive cycles (without first heating) up to 875 °C: electrical conductivity σ (top, left), Seebeck coefficient S (top, right), thermal conductivity κ (middle, left), power factor PF (middle, right) and thermoelectric figure of merit ZT (bottom, left).

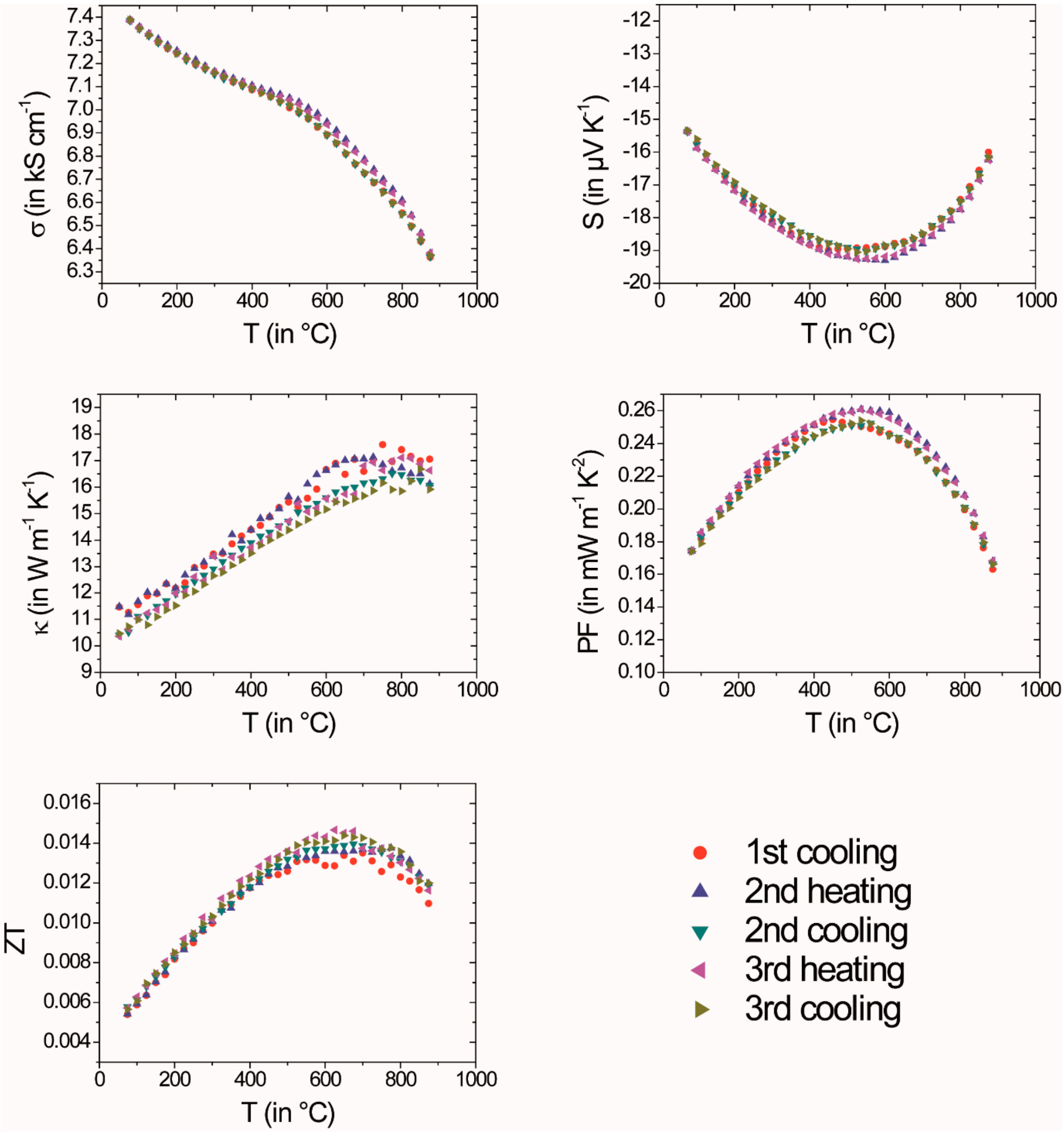

Figure S14. Thermoelectric properties of $Al_2CrCoFeNi$ + 0.3 w% Sc for three consecutive cycles (without first heating) up to 875 °C: electrical conductivity σ (top, left), Seebeck coefficient S (top, right), thermal conductivity (middle, left), power factor PF (middle, right) and thermoelectric figure of merit ZT (bottom, left).

References

1. Norris, S.D., Parker, J.D., Deformation processes during disc bend loading, *Mat. Sci. Tecnol.* **1996**, *12*, 163-170.
2. Lacalle, R., Alvarez, J.A., Gutiérrez-Solana, F., Analysis of key factors for the interpretation of small punch test results, *Fatigue Fract. of Eng. Mat Struct.* **2008**, *31*, 841-849.
3. Hurst, R., The european code of practice for small punch testing: where do we go from here?, *Metall. J.* **2010**, *63*, 5-11.
4. Džugan, J., Konopìk, P., Evaluation of fracture toughness properties for low carbon steel in the brittle state by small punch test technique, *Metall. J.* **2010**, *63*, 119-122.
